# Accelerated Development of Vapor Deposition Technology for Efficient Perovskite Solar Cells via Accurate and Practical Machine Learning Tools

**DOI:** 10.1002/advs.202510946

**Published:** 2025-08-13

**Authors:** Long Luo, Ziyang Gao, Muyi Fang, Yu Shen, Shuaizhang Chen, Yuchen Bu, Junjie Gu, Cheng Hu, Jianning Ding

**Affiliations:** ^1^ College of Physical Science and Technology Institute of Technology for Carbon Neutralization, Yangzhou University Jiangsu 225009 China; ^2^ Business School Institute of Technology for Carbon Neutralization Yangzhou University Jiangsu 225009 China; ^3^ College of Chemical and Environmental Engineering Wuhan Polytechnic University Hubei 430000 China

**Keywords:** Machine learning, Perovskite solar cells, Vapor deposition technology

## Abstract

The development of vapor deposition technology will accelerate the process of perovskite solar cells (PSCs) moving from laboratory scale to industrialization. However, the multi‐dimensional and complex parameter space will inevitably increase the cost of trial and error, especially for high‐energy‐consuming and long‐cycle vapor deposition technologies. This study employed an integrated‐feature dataset encompassing macro‐ and micro‐features to enhance the accuracy, robustness, and interpretability of an ETree machine learning (ML) model for power conversion efficiency (PCE) prediction, achieving an impressive coefficient of determination value of 0.9464 and root mean square error value of 1.27%. Through SHAP analysis, Monte Carlo simulations, and parameter space exploration, an optimal FTO/SnO_2_/Cs_0.04_FA_0.96_PbI_3_/Spiro‐OMeTAD/Au device architecture and vapor deposition parameters are reverse‐engineered, yielding the highest predicted PCE of 26.21%. Furthermore, the PCEs are enhanced by implementing the universal ML‐derived optimization strategies across six distinct and independent vapor deposition processing, which truly realizing the objective of ML‐guided experiments based on various preparation conditions. This machine learning model is believed to shorten the research and development cycle for breaking through the performance bottleneck of high‐efficiency devices fabricated by vapor deposition technology, providing a potential approach for its commercial application.

## Graphical abstract

Based on rational feature selection, an integrated‐feature dataset is constructed encompassing macro‐ and micro‐feature parameters. Screening and optimization across ten algorithmic models enhanced predictive accuracy and robustness for power conversion efficiency (PCE), yielding an *R* ^2^ of 0.9464 and RMSE of 1.27%. Based on these optimized parameters, combining SHAP analysis, Monte Carlo simulations, and parameter space exploration determined an optimal FTO/SnO_2_/Cs_0.04_FA_0.96_PbI_3_/Spiro‐OMeTAD/Au architecture with vapor deposition parameters. Implementing these ML‐derived strategies across six vapor deposition techniques achieved the highest predicted PCE of 26.21%. This establishes a closed‐loop framework integrating comprehensive feature selection, predictive accuracy, and model interpretability with practical utility, providing actionable guidance for vapor‐deposited high‐efficiency perovskite solar cells.

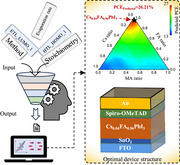

## 1 Introduction

Through rational materials design and innovative processing techniques, the certified power conversion efficiencies (PCEs) of state‐of‐the‐art organic‐inorganic hybrid perovskite solar cells (PSCs) have achieved over 27%.^[^ ^1^ ^]^ In the conventional sandwich‐type device architecture, the perovskite photoactive layer can be fabricated via either solution‐processing or vapor‐phase deposition routes.^[^ ^2^, ^3^, ^4^ ^]^ Although solution deposition technology is limited by solvent toxicity concerns and difficulties in controlling crystallization kinetics over large areas, they are still favored by both academia and industry due to its advantages such as diverse regulation techniques, high deposition rates, and low material and equipment costs.^[^ ^5^, ^6^ ^]^ In contrast, vapor deposition methods provide superior film uniformity, solvent‐free processing, and better compatibility with existing photovoltaic manufacturing infrastructure.^[^ ^7^, ^8^, ^9^ ^]^ Nevertheless, these vacuum‐based techniques present their own set of limitations, including lower deposition rates, higher precursor utilization costs, and more complex process parameter optimization requirements ‐ all of which contribute to extended R&D cycles and substantial capital investment.^[^ ^10^, ^11^ ^]^ The quickly increased PCE of vapor‐deposited PSCs highlight the tremendous potential of this fabrication route.^[^ ^3^, ^12^ ^]^ However, the multidimensional parameter space encompassing precursor chemistry and the intricate nature of carrier transport materials would inevitably result into the labor‐extensive trial‐and‐error efforts to overcome current efficiency bottlenecks and approach the theoretical PCE limit of 33%, especially for vapor deposition technology.^[^ ^13^, ^14^, ^15^ ^]^ Therefore, it is urgent to accelerate the research on vapor deposition technology to promote its application and development in industrialization.

In recent years, machine learning (ML) has emerged as a powerful tool for accelerating materials discovery and optimizing device performance in photovoltaics.^[^ ^16^, ^17^, ^18^, ^19^ ^]^ While ML models incorporating electronic structure features (e.g., energy levels, carrier mobilities),^[^ ^20^, ^21^ ^]^ molecular‐scale features,^[^ ^22^, ^23^ ^]^ and thin‐film optoelectronic properties (e.g., fluorescence characteristics)^[^ ^24^, ^25^ ^]^ have demonstrated reasonable predictive accuracy, these microscopic parameters often lack direct experimental applicability due to challenges in precise measurement and process control. Moreover, attempting to predict PCE based solely on macroscopic device architectures, material selections, and fabrication methods similarly yield unsatisfactory results and lack robustness. The end‐to‐end mapping models trained solely on these macro‐feature parameters are highly susceptible to limitations in training data scope and quality. Such models are prone to overfitting, exhibit poor extrapolation capability, and demonstrate limited utility for guiding practical materials exploration (shown in Table S1, Supporting Information). Therefore, improving model robustness, interpretability, and practicality still be an urgent challenge.

Herein, based on rational and comprehensive feature selection, we first constructed an integrated‐feature dataset encompassing macro‐ and micro‐feature parameters. Subsequent screening and optimization across 10 algorithmic models enhanced predictive accuracy and robustness for PCE, yielding a coefficient of determination (*R* ^2^) of 0.9464 and root mean square error (RMSE) of 1.27%. Based on these optimized model parameters, we further reverse‐engineered an optimal FTO/SnO_2_/Cs_0.04_FA_0.96_PbI_3_/Spiro‐OMeTAD/Au device architecture with vapor deposition parameters via SHapley Additive exPlanations (SHAP) analysis, Monte Carlo simulations, and parameter space exploration methods. Finally, we achieved enhancements in PCE by implementing the ML‐derived optimization strategies in six distinct vapor deposition processes. Consequently, this research process establishes a closed‐loop framework integrating comprehensive feature selection, predictive accuracy, and model interpretability with practical utility, which provides practical guidance for the application of vapor deposition technology in fabricating high‐efficiency PSCs.

## 2 Results and Discussion

### 2.1 Workflow and Dataset Construction

**Figure 1:**
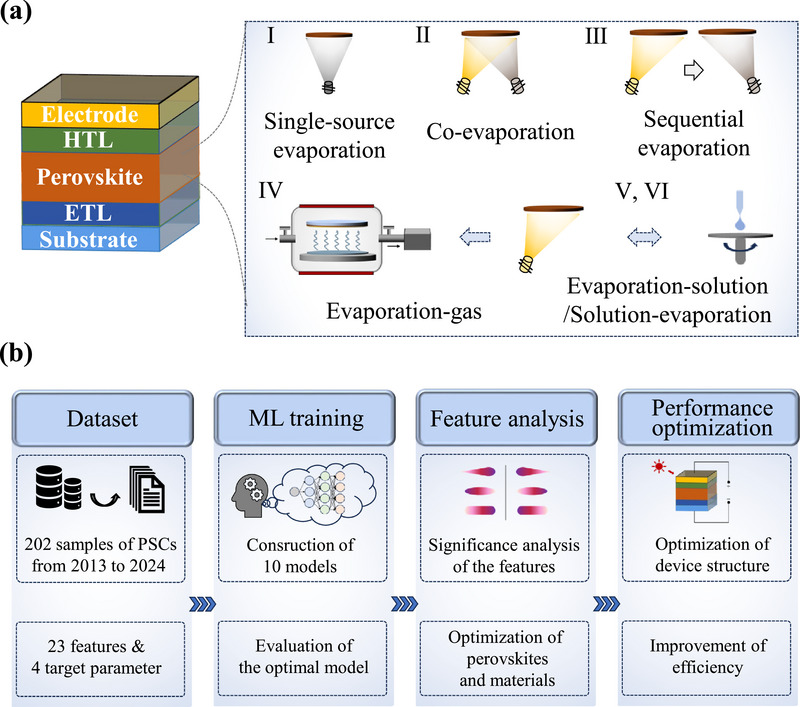
Workflow of accelerating the development of vapor deposition technology for efficient PSCs. a) Device structure of PSCs and six perovskite thin film vapor deposition techniques. b) Schematic diagram of the ML workflow for vapor‐deposited efficient PSCs.

As illustrated in **Figure** **1**a, PSCs exhibit a classical layered architecture comprising five critical components: the substrate, electron transport layers (ETLs), perovskite absorber layer, hole transport layers (HTLs), and electrode. Among them, the stoichiometric precision and crystallization kinetics behaviors of perovskite films often profoundly affect the photovoltaic performance of the devices, which promotes the development of six distinct vapor deposition techniques to achieve an optimal balance between stoichiometric precision and crystallization control. However, the intricate interplay between multilayer materials and complex deposition parameters increases traditional trial‐and‐error approaches costs and time‐consuming for technological advancement. To address this challenge, we have developed a convincing ML framework specifically tailored for vapor deposition optimization (Figure 1b). First, a set of 202 date points was extracted from peer‐reviewed articles spanning 2013–2024, followed by rigorous data preprocessing including cleaning, missing value imputation, and augmentation (shown in Table S2, Supporting Information). The input feature space encompassed 23 carefully selected experimental parameters, categorized into three fundamental aspects: 1) material composition (cation/anion types and stoichiometric ratios in the perovskite), 2) material properties (substrate/electrode work functions, HOMO/LUMO levels of charge transport layers), and 3) fabrication parameters (encompassing all six vapor deposition method variants I‐VI and their associated processing conditions such as AX/BX_2_ deposition rate, chamber pressure, annealing temperature and time, and device area) (shown in Table S3, Supporting Information). These input features were encoded, with particular attention given to proper representation of categorical variables through one‐hot encoding. The output variables consisted of four photovoltaic characteristics were open‐circuit voltage (*V*_OC_), short‐circuit current density (*J*_SC_), fill factor (FF), and PCE. Comprehensive correlation analysis via Pearson coefficients (|*p*|<0.8 for most variable pairs, Figure S1, Supporting Information) confirmed the absence of significant feature redundancy, while intentionally retaining certain correlated parameters (e.g., ETL/HTL bilayer structure and anion ratios) to enable investigation of the practical structure‐property relationships.^[^ ^26^, ^27^ ^]^

### 2.2 Machine Learning Model Training

**Figure 2:**
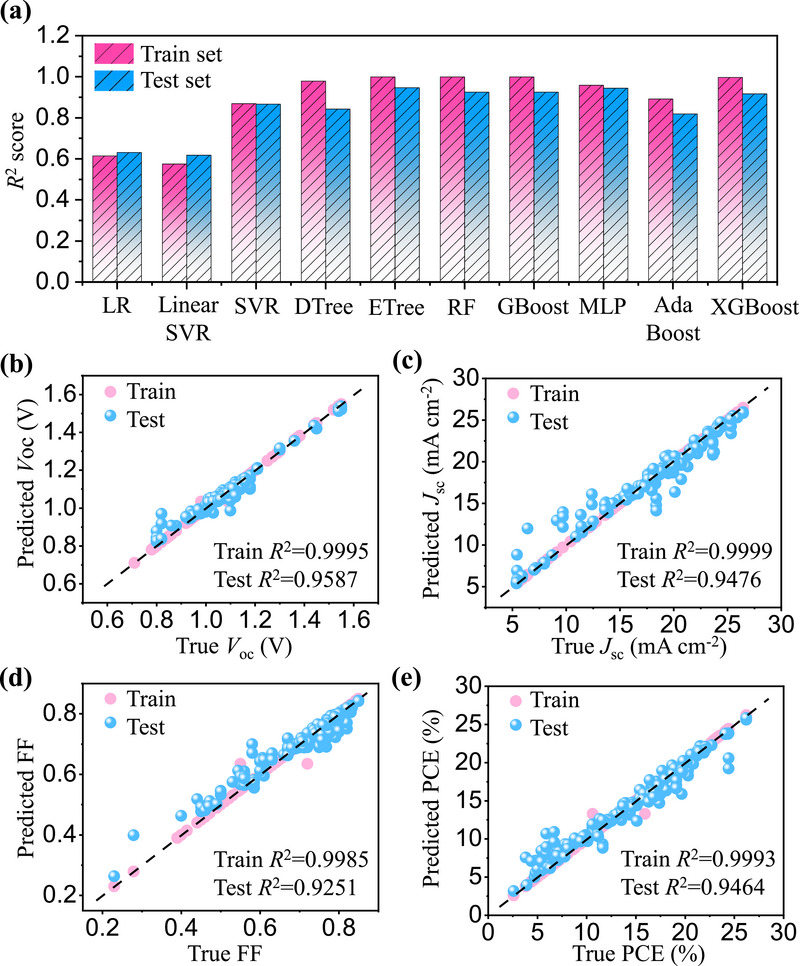
Evaluation of ML models. a) *R* ^2^ value histograms of 10 ML models for PCE prediction. b–e) The fitting graph of *V*_OC_, *J*_SC_, FF and PCE results by the ETree model, where blue represents the training set and pink represents the test set.

For predictive modeling, we systematically evaluated ten ML algorithms encompassing both classical and advanced approaches: linear regression (LR),^[^ ^28^ ^]^ linear support vector regression (linear SVR),^[^ ^29^ ^]^ support vector regression (SVR),^[^ ^30^ ^]^ decision tree (DTree),^[^ ^31^ ^]^ extra trees (ETree),^[^ ^32^ ^]^ random forest (RF),^[^ ^33^ ^]^ gradient boosting (GBoost),^[^ ^34^ ^]^ multilayer perceptron (MLP),^[^ ^35^ ^]^ Adaptive Boosting (AdaBoost),^[^ ^36^ ^]^ and extreme gradient boosting (XGBoost).^[^ ^37^ ^]^ The dataset was randomly divided into a training set (80% of the data) and a test set (the remaining 20%). The rigorous hyperparameter optimization was performed via grid search coupled with cross‐validation to ensure model generalizability (Table S4, Supporting Information). To comprehensively assess the predictive ability and accuracy of the model, three evaluation criteria were employed: the coefficient of determination (*R* ^2^) for goodness‐of‐fit, the root mean square error (RMSE) for absolute error magnitude, and the mean absolute percentage error (MAPE) for relative error. The model fitting status across both training and test sets was visually presented in **Figure** **2**b–e and Figures S2–S11 (Supporting Information), with consolidated predictive capabilities summarized in Figure 2a and Figure S12 and Table S5 (Supporting Information). Notably, all models demonstrated consistent performance between training and test sets without evidence of overfitting. Among the evaluated algorithms, the ETree, RF, GBoost, MLP, and XGBoost models exhibited excellent performance for PCE prediction, with *R* ^2^ values exceeding 0.9 and lower RMSE and MAPE values. The ETree model achieved best predictive accuracy for PCE with an *R* ^2^ of 0.9464, RMSE of 1.273%, and MAPE of 8.318% on the test set. Equally impressive performance was observed for other photovoltaic parameters (*V*_OC_, *J*_SC_, and FF) that they all maintained *R* ^2^ > 0.9, thereby confirming a strong correlation between predicted and experimental values across all key performance parameters (method details in Code description section of Supplementary Materials). The superior predictive performance of our machine learning model primarily stems from the comprehensive feature dataset and the distinctive advantages of the ETree algorithm. As an ensemble learning method, the ETree algorithm enhances decision trees by introducing extreme randomness in feature selection and split point determination during tree construction.^[^ ^38^ ^]^ This randomization effectively reduces overfitting and variance, which is crucial for model predictions based on datasets integrating macro‐ and micro‐feature parameters. Therefore, Etree model could balance bias and variance efficiently so that show better performance compared to other algorithms.^[^ ^39^, ^40^ ^]^ Consequently, our ML framework could achieve remarkable predictive accuracy while maintaining robustness against overfitting in this complex materials system.

### 2.3 Model Explanation

**Figure 3:**
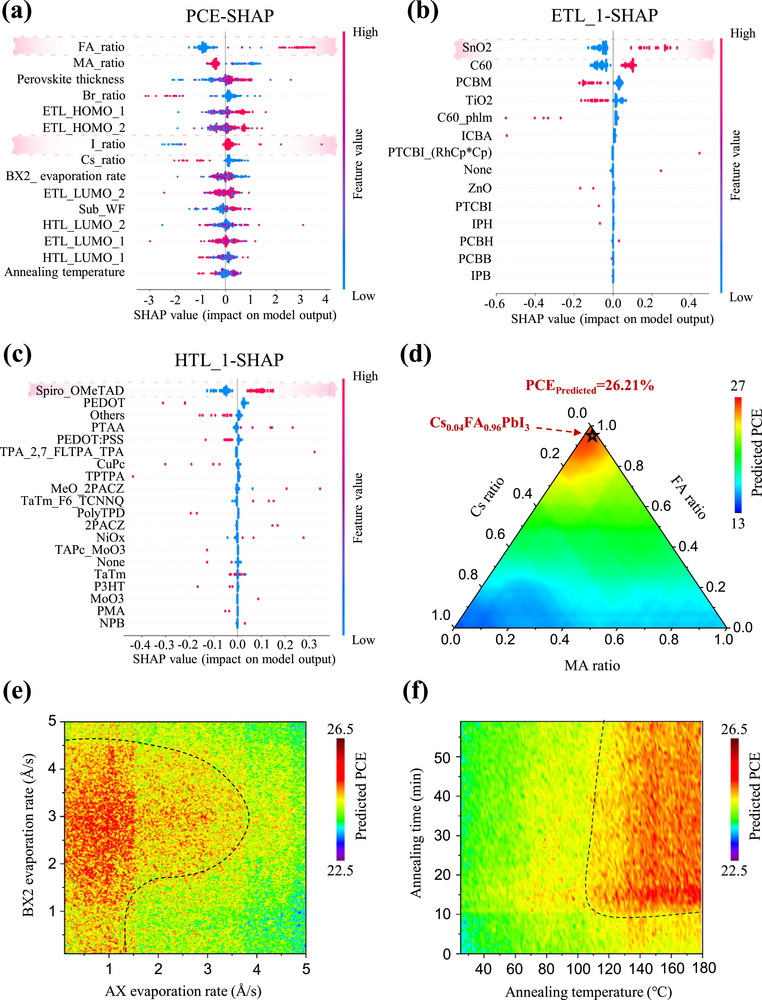
The contribution of different influencing factors to PCE. The SHAP analysis result graph for all variables a), ETL_1 b) and HTL_1 c). d) Triangular heat map of the effects of MA, FA, and Cs cations on PCE based on the sequential evaporation deposition method. The heat map obtained through the Monte Carlo simulation method shows the influence of the deposition rate of the perovskite precursor e) and the annealing conditions f) during the sequential evaporation deposition process on the device efficiency.

The SHAP method was utilized to interpret the model and assess the significance of various input features in determining photovoltaic performance. Six high‐performing predictive models were selected for cross‐validation to evaluate the positive or negative contributions of these features to PCE (**Figure** **3**a; Figures S13–S15, Supporting Information). In the SHAP plots, each row represents a feature ranked by descending importance. Individual data points correspond to samples. The horizontal axis denotes the SHAP values, where positive values indicate favorable contributions to PCE, while negative values suggest detrimental effects. Red and blue points signify high and low feature values, respectively. SHAP analysis results based on the ETree model in Figure 3a revealed that among the top 15 influential variables, perovskite composition‐related parameters dominated, demonstrating the critical role of perovskite materials in device performance (method details in Code description section of Supplementary Materials). Specifically, a higher formamidinium (FA) ratio exhibited a strong positive correlation with PCE, and certain low‐FA‐ratio samples (blue points) displayed negative SHAP values. Furthermore, a high iodine (I) ratio and low bromine (Br) ratio enhanced PCE. These trends were also proved by SHAP analyses from four additional models (Adaboost, DTree, GBoost, and XGBoost) (Figure S13, Supporting Information), reinforcing the robustness of our findings. Expanding the analysis to other photovoltaic parameters (*V*_OC_, *J*_SC_, and FF) using ETree and RF models, we observed that increasing the Br ratio significantly improved *V*_OC_ but detrimentally affected *J*_SC_ (Figures S14 and S15, Supporting Information)_._ Regarding the SHAP analysis results, we analyze them from perspectives of material intrinsic properties and vapor deposition applications. First, compared to pure MA‐based or Cs‐based perovskite materials, FA‐based perovskites exhibit narrower bandgaps and broader spectral response ranges.^[^ ^41^, ^42^, ^43^ ^]^ Similarly, increased iodine ratio among halide anions reduces perovskite bandgaps, enabling enhanced light harvesting that substantially boosts short‐circuit current in devices.^[^ ^44^ ^]^ Microstructurally, methylammonium (MA)‐based perovskites form dense, anisotropic dynamic nanodomains featuring antiphasic octahedral tilting, whereas formamidinium (FA)‐based systems develop sparse, isotropic spherical nanodomains exhibiting in‐phase tilting. These sparsely distributed spherical nanodomains reduce electron dynamic disorder, thereby optimizing photoresponse.^[^ ^45^ ^]^ Consequently, FAPbI_3_ perovskite with its optimal bandgap achieves a theoretical efficiency limit exceeding 31%, emerging as the most promising lead‐based perovskite material.^[^ ^46^, ^47^ ^]^ However, the metastable nature at room temperature and moisture sensitivity of FAPbI_3_ facilitate its transition to the optically inactive yellow phase, preventing pure FAPbI_3_ PSCs from achieving expected performance.^[^ ^48^, ^49^ ^]^ Therefore, to enhance FA‐based perovskite stability, researchers are compelled to use the multicomponent cation‐anion mixing perovskite system through compositional engineering at the expense of sacrificing their potential performance.^[^ ^50^ ^]^ Regrettably, high‐quality mixed‐ion perovskite film deposition remains challenging due to poor stoichiometric control in vapor‐deposited films and crystallization processes influenced by interactions with underlying charge‐transport materials. Consequently, near‐phase‐pure FAPbI_3_ perovskite persists as the prime light absorbers candidate for efficient, stable vapor‐deposition‐processed PSCs. Detailed analysis follows in subsequent sections.

To gain deeper insights into the impact of functional layer materials on device performance and provide practical experimental guidance, we performed SHAP analysis on method, substrate, ETLs, perovskite, HTLs, and back electrode variables using the ETree model. These categorical variables were processed via one‐hot encoding. First, the SHAP results (Figure S16a, Supporting Information) demonstrated that while co‐evaporation deposition method remained prevalent, the highest‐performing devices predominantly employed two‐step deposition techniques such as evaporation‐gas deposition, evaporation‐solution processing, and sequential evaporation deposition. This might mainly be due to the difference in vapor partial pressure between organic and inorganic components during the co‐evaporation process, which makes the film crystallization process difficult to control.^[^ ^9^ ^]^ However, these two‐step vapor deposition techniques could more effectively control the diffusion behavior of organic components in inorganic components, which is conducive to the formation of high‐quality films. Regarding ETL and HTL, the SHAP results (Figure 3b,c; Figure S16b,c, Supporting Information) showed that SnO_2_ and Spiro‐OMeTAD represented optimal choices for electron and hole transport materials in high‐efficiency vapor‐deposited PSCs, without requiring additional modification or composite layers. Actually, the properties of underlying charge‐transport materials significantly influence crystallization during subsequent thermal‐evaporation deposition of lead iodide or perovskite films.^[^ ^11^, ^51^ ^]^ Amorphous materials like C₆₀, PCBM, and PTAA might induce parallel substrate‐aligned growth of dense lead iodide films due to isotropic surface energy, whereas polycrystalline SnO_2_ promotes tilted/vertical growth of loosely porous structures via anisotropic surface energy, facilitating organic ammonium salt infiltration. Furthermore, the formation of perovskite films requires sustained high temperatures (≥160 °C) in this two‐step vapor deposition process, demanding superior thermal stability from bottom charge‐transport layers.^[^ ^52^, ^53^ ^]^ Consequently, polycrystalline SnO_2_ with high transmittance, conductivity, and thermal stability becomes the optimal electron transport material for vapor‐deposition‐processed devices. Meanwhile, Spiro‐OMeTAD material with excellent film‐forming capability and hole‐extraction efficiency naturally serves as the preferred hole transport material for n‐i‐p architectures.^[^ ^54^, ^55^ ^]^ Substrate and electrode materials exhibited relatively minor influence in vapor‐deposited devices (Figure S16d,e, Supporting Information). For the critical perovskite composition, we conducted a comprehensive parameter space exploration of FA, MA, Cs, Br, and I ratios to obtain the potential optimal perovskite stoichiometric ratio. Based on the existing SHAP analysis results, we first determined optimal functional materials to derive the FTO/SnO_2_/Perovskite/Spiro‐OMeTAD/Au device architecture. Subsequently fixing these relevant feature parameters, we employed Monte Carlo simulations and perovskite composition grid‐based searches to iteratively simulate sequential vapor deposition processes. This approach ultimately predicted optimal perovskite compositions, associated vapor deposition processing parameters and the corresponded highest PCE value (method details in Code description section of Supplementary Materials). It could be seen from the Figure S17 (Supporting Information) that as the I ratio increased, the PCE of the device showed an almost linear growth trend. When the I ratio reached 1, the predicted efficiency of device reached at the maximum value. As for the cation ratio, the optimal ratio occurred at the parameter spatial position of MA: FA: Cs = 0: 0.96: 0.04. Ultimately, we discovered that the optimal perovskite composition was Cs_0.04_FA_0.96_PbI_3_, and the predicted efficiency of the devices fabricated from this was as high as 26.21%. The above SHAP analysis revealed that while phase‐pure FAPbI_3_ represented theoretically optimal stoichiometry, its metastability at room temperature and tendency to form supersaturated‐phase impurities during vapor‐solid reactions limited practical vapor‐deposition processing.^[^ ^7^, ^56^ ^]^ Although incorporating smaller MA/Cs cations or Br anions enhanced FA‐based perovskite stability, increased bandgap and indeterminate stoichiometry (typically formulated as Cs_x_MA_y_FA₁₋_x_₋_y_PbI_3_₋_z_Br_z_) constrained device performance and reproducibility. To address this, researchers pre‐mixed CsI/PbI_2_ powders into molten salts or thermally evaporated CsI/PbI_2_ composite films, subsequently reacting with FAI vapor at defined Cs_x_PbI_2_₊_x_ stoichiometry to form precisely controlled Cs_x_FA₁₋_x_PbI_3_ films, which enhanced the efficiency and stability of devices.^[^ ^57^, ^58^, ^59^ ^]^ Furthermore, cesium halides promoted organic ammonium salt diffusion during vapor‐solid reactions, inducing uniform crystallization and suppressing PbI_2_/yellow‐phase residues.^[^ ^60^, ^61^ ^]^ Thus, the predicted CsFA composition is validated. Additionally, we further analyzed the effects of perovskite precursor deposition rate and annealing parameter on PCE values in sequential vapor deposition via Monte Carlo simulations (Figure 3e,f). Among 500 000 simulated annealing‐efficiency related data points, optimal efficiencies clustered at the region between low AX deposition rates and moderate BX_2_ rates. This is mainly because when the deposition rate of the organic ammonium salt AX is relatively fast, its large gas pressure makes it difficult to control the crystallization of the perovskite.^[^ ^11^ ^]^ On the contrary, the more stable inorganic lead halide BX2 can be deposited at a moderate deposition rate, which can make the film more porous and loose, facilitating the penetration of subsequent organic ammonium gas.^[^ ^62^ ^]^ Finally, sustained higher‐temperature annealing simultaneously removes excess organic salts at the perovskite surface and promotes perovskite grain growth, which therefore improved PSC efficiency.

### 2.4 Model Prediction and Potential Optimization Strategies

To verify model robustness and accuracy, we first demonstrated dataset representativeness. We incorporated the eleven additional data points into the original test set and used pre‐optimized model parameters to predict efficiency (Table S6, Supporting Information). The *R* ^2^ value of the prediction model trained by the new test dataset maintained 0.94 and obtained the RMSE and MAPE value comparable to the original model performance parameters, which confirmed our dataset's relatively strong representativeness (Figure S18 and Table S7, Supporting Information). To demonstrate the predictive accuracy of the model, we evaluated the deviation between predicted PCE (PCE_predicted_) and experimentally measured PCE (PCE_real_) via using recently published experimental data as new input parameters (method details in Code description section of Supplementary Materials). As demonstrated in Figure S19 (Supporting Information), we analyzed three distinct vapor‐deposited device architectures and their corresponding PCE value. The model exhibited remarkable predictive precision with an error of less than 4.45% between PCE_predicted_ values and PCE_real_ values. Among them, the PCE_predicted_ value of the PSC with ITO/MeO‐2PACz/Cs_0.2_FA_0.94_Pb(I_0.94_Br_0.11_)_3_/C_60_/BCP/Ag structure based on co‐evaporation technology differed from the PCE_real_ value by only 0.08, and the error is close to 0.4% (Figure S18a, Supporting Information).

**Figure 4:**
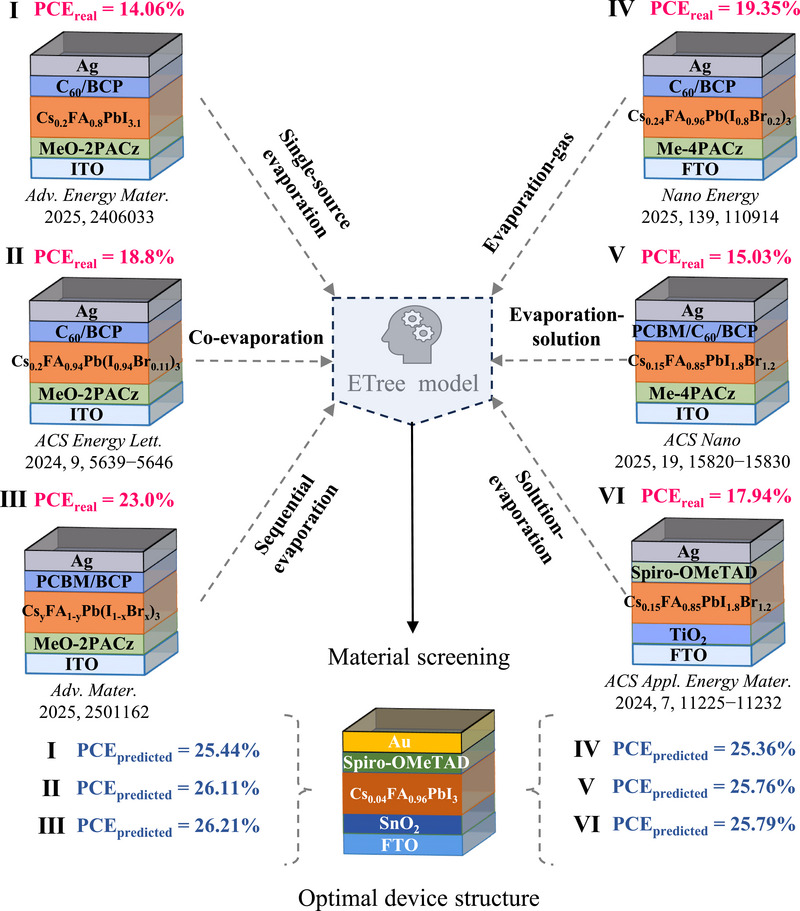
Device structure optimization and PCE enhancement based on six different vapor deposition techniques.

For each specific vapor deposition method, the potential PCE can be predicted by optimizing the stoichiometry of perovskite, the structure of the device, and the processing techniques. As shown in **Figure** **4**, we focused on six prevalent vapor deposition methods (I‐IV) and obtained their device structures and tested PCE values. It could be found that their PCEs were generally low at present, which was also agreeable with the current development status of vapor deposition technology. We further determined the optimal Cs_0.04_FA_0.96_PbI_3_ perovskite compositions, FTO/SnO_2_/Cs_0.04_FA_0.96_PbI_3_/Spiro‐OMeTAD/Au device architectures, and corresponding processing parameters for each of the six vapor‐deposition techniques. After prediction through an ML model, we found that the predicted PCE of the improved devices based on six different vapor deposition techniques was enhanced. Most notably, sequential evaporation deposition and co‐evaporation deposition demonstrated remarkable potential with a predicted PCE up to 26.21% and 26.11%, respectively. These findings not only validate the predictive accuracy of our ML framework but also establish a robust materials optimization strategy that bridges fundamental research with industrial‐scale manufacturing considerations for vapor‐deposited PSCs.

## 3 Conclusion

Through comprehensive data collection of over 136 publications, we constructed a dataset comprising 202 data points with 23 carefully selected input features. Based on this, we developed a robust ML model demonstrating strong predictive performance on the test set: with a high *R* ^2^ value of 0.9464, coupled with low RMSE (1.273%) and MAPE (8.318%) values. Our systematic analysis had not only identified key performance‐limiting factors but also yielded specific optimization strategies tailored to different vapor deposition methods. The model revealed that SnO_2_ as the electron transport layer and Spiro‐OMeTAD as the hole transport layer exhibited particularly strong positive correlations with enhanced device performance. Most importantly, we further reverse‐engineered an optimal FTO/SnO_2_/Cs_0.04_FA_0.96_PbI_3_/Spiro‐OMeTAD/Au device architecture with vapor deposition parameters via SHapley Additive exPlanations (SHAP) analysis, Monte Carlo simulations, and parameter space exploration methods. The predicted PCE of devices achieved remarkable efficiencies up to 26.21%. At last, in order to validate the practical applicability of our findings, we selected the latest advanced devices fabricated by six different vapor deposition techniques. We optimized from the most intuitive device structure and vapor deposition parameters, which significantly improved the prediction efficiency of the improved devices. This research establishes a powerful ML framework to explore the fabrication of efficient PSCs based on vapor deposition technologies, which might accelerate the industrialization of perovskite photovoltaic technology.

## Conflict of Interest

The authors declare no conflict of interest.

## Author Contributions

L.L. and C.H. conceived and supervised the project. J.D. directed the research. L.L. and C.H. conducted the training and analysis of machine learning models. Z.G., M.F., Y.B., J.G., Y.S., and S.C. carried out the data collection and analysis. L.L. drafted the manuscript. All authors revised the manuscript.

## Supporting information



Supporting Information

## Data Availability

The data that support the findings of this study are available from the corresponding author upon reasonable request.
