# Supplementary material for: Accelerated Development of Vapor Deposition Technology for Efficient Perovskite Solar Cells via Accurate and Practical Machine Learning Tools
Source: Adv Sci (Weinh). 2025 Aug 13;12(42):e10946. doi: 10.1002/advs.202510946 (PMC12622531; doi:10.1002/advs.202510946)
Supplement: Supplementary file 1 — Supporting Information [file ADVS-12-e10946-s001.docx]

Supporting Information

**Accelerated Development of Vapor Deposition Technology for Efficient Perovskite Solar Cells via Accurate and Practical Machine Larning Tools**

*Long Luo*^1,^**, Ziyang Gao*^3^*, Muyi Fang*^1^*, Yu Shen*^1^*, Shuaizhang Chen*^1^*, Yuchen Bu*^1^*, Junjie Gu*^1^*, Cheng Hu*^2,^**, and Jianning Ding*^1^

^1^L. Luo, M. Fang, Y. Shen, S. Chen, Y. Bu, J. Gu, Prof. J. Ding

College of Physical Science and Technology, Institute of Technology for Carbon Neutralization

Yangzhou University

Jiangsu 225009, China

^2^Dr. C. Hu

College of Business, Institute of Technology for Carbon Neutralization

Yangzhou University

Jiangsu 225009, China

^3^Z. Gao

College of Chemical and Environmental Engineering

Wuhan Polytechnic University

Hubei 430000, China

**Corresponding author**

*E-mail: hucheng@yzu.edu.cn

*E-mail: longluo@yzu.edu.cn

***Dataset construction***

We cleared the data points of non-lead-based perovskite devices and perovskite stack devices contained in the original dataset. Consequently, the dataset for ML was composed of 202 data points from 136 articles published between 2013 and 2024. The detailed data are shown in **Table S1**. We further expanded the dataset fivefold to over 1000 data points using Gaussian noise. This data augmentation *via* Gaussian noise introduction effectively enhanced data diversity, thereby improving model generalization capability and mitigating limitations from small sample size.^[1]^

Based on the mode of vapor deposition, the techniques in the dataset are categorized into six types: single-source evaporation, co-evaporation, sequential evaporation, evaporation-gas, evaporation-solution, and solution-evaporation.^[2, 3]^ Their brief descriptions are as follows: (1) Single-source evaporation primarily includes single-source thermal evaporation deposition and single-source pulsed laser vapor deposition, wherein the latter involves laser photons interacting with the target material by exciting bound electrons, followed by energy transfer to the material's lattice via electron-phonon coupling to promote thermalization; (2) Co-evaporation mainly refers to dual-source or multi-source thermal co-evaporation deposition; (3) Sequential evaporation primarily is multi-step thermal evaporation depositions; (4) Evaporation-vapor combines thermal evaporation of inorganic lead halides with low-pressure vapor deposition techniques such as chemical vapor deposition (CVD) or close-space sublimation of organic ammonium salts in a two-step deposition process; (5) Evaporation-solution mainly involves a two-step deposition process starting with thermal evaporation deposition followed by solution reaction; (6) Solution-evaporation primarily consists of a two-step deposition process beginning with solution deposition followed by thermal evaporation or CVD reaction.

***Model selection***

**Linear regression (LR)** **algorithm:** LR is a fundamental algorithm for prediction by establishing a linear relationship equation between the independent variable and the continuous dependent variable. This method emphasizes the global linear assumption and model interpretability - the coefficients directly reflect the direction and intensity of the influence of features on the target variable, but there are obvious limitations: it cannot capture nonlinear relationships and is sensitive to outliers.

**Support vector regression (SVR) algorithm:** SVR identifies an optimal hyperplane that fits training data while preserving generalization capability. Distinctively, SVR employs an ε-insensitive tube around the predicted function, where errors within the tube incur no penalty. Only support vectors outside this tube contribute to the loss function. Optimization minimizes empirical error while maximizing the margin, balancing model complexity and predictive accuracy.

**Linear** **support vector regression (Linear SVR)** **algorithm:** As a specialized SVR variant, Linear SVR prioritizes computational efficiency for linear relationships by restricting itself to linear kernels. While incapable of modeling nonlinear patterns, it eliminates kernel selection and hyperparameter tuning overhead. This results in significantly accelerated training and inference compared to kernel-based SVR.

**Decision tree (DTree)** **algorithm:** DTree recursively partitions the feature space through binary splits, forming an interpretable hierarchical structure. At each internal node, a feature is selected based on maximum information gain, while terminal nodes represent class labels or regression values.

**Random forest (RF)** **algorithm:** RF is a bagging-based ensemble method for classification and regression tasks. It constructs diverse decision trees by training each on bootstrapped data subset with random feature selection at node splits. Final prediction aggregate outputs via majority voting (classification) or averaging (regression), enhancing robustness and mitigating overfitting.

**Extra trees (ETree)** **algorithm:** ETree extends bagging with extreme randomization. Unlike RF, it uses the full training set with random feature subsets, and selects split points randomly. This maximizes tree decorrelation, often improving generalization and training speed.

**Multilayer perceptron (MLP)** **algorithm:** MLP is a deep learning architecture of interconnected perceptron, loosely simulating biological neurons. Comprising input, hidden, and output layers, it propagates features forward through weighted transformations and nonlinear activations. Backpropagation optimizes parameters (weights/biases) via chain-rule gradient computation to minimize loss functions. This hierarchical structure enables universal approximation of complex input-output mappings.

**Gradient boosting (GBoost)** **algorithm:** GBoost iteratively builds an ensemble of weak learners (typically trees) to form a strong predictor. At each iteration, a new tree fits the negative gradient (pseudo-residuals) of the current ensemble’s loss function, functionally optimizing in hypothesis space. Subsequent trees focus on correcting predecessors’ errors, progressively reducing residual error.

**Adaptive boost (AdaBoost)** **algorithm:** AdaBoost adaptively reweights samples to prioritize instances misclassified by previous weak learners. Each iteration increases weights for mispredicted samples and decreases weights for correct predictions, forcing subsequent classifiers to focus on challenging cases. Predictions are combined via weighted majority voting until convergence or maximum iterations.

**Extreme gradient boosting (XGBoost)** **algorithm:** XGBoost enhances traditional gradient boosting through formalized optimization. While both methods iteratively minimize residuals, XGBoost supports custom loss functions requiring first and second derivatives, enabling flexible regularization. Its framework incorporates second-order gradient information for precise optimization and includes built-in regularization to control complexity.

***Model evaluation***

**Coefficient of determination (*R*^2^):** *R*² quantifies the proportion of variance in the dependent variable explainable by independent variables, serving as a fundamental metric for assessing regression model performance. Ranging from 0 to 1, it measures model fit quality, where values approaching 1 indicate high explanatory power and values near 0 suggest poor predictability.

**Root mean square error (RMSE):** RMSE measures prediction accuracy as the square root of average squared prediction errors. As a scale-dependent metric, it reflects absolute error magnitude, with lower values indicating higher precision and superior model performance.

**Mean absolute percentage error (MAPE):** MAPE expresses average absolute prediction error as a percentage relatively to actual values. It weights all errors equally, providing an intuitive and interpretable accuracy measure. Notably, MAPE is sensitive to near-zero actual values and may exhibit bias.

***Code abstract***

**Train of Etree model:**

import pandas as pd

import numpy as np

import matplotlib.pyplot as plt

from sklearn.compose import ColumnTransformer

from sklearn.preprocessing import OneHotEncoder, StandardScaler

from sklearn.pipeline import Pipeline

from sklearn.ensemble import ExtraTreesRegressor

from sklearn.model_selection import GridSearchCV, KFold

from sklearn.metrics import mean_squared_error, r2_score

train_df = pd.read_excel('train set.xlsx')

test_df = pd.read_excel('test set.xlsx')

X_train = train_df.iloc[:, 4:]

y_train = train_df.iloc[:, :4]

X_test = test_df.iloc[:, 4:]

y_test = test_df.iloc[:, :4]

categorical_cols = [0]

numerical_cols = list(range(1, X_train.shape[1]))

preprocessor = ColumnTransformer(

transformers=[

('cat', OneHotEncoder(handle_unknown='ignore'), categorical_cols),

('num', StandardScaler(), numerical_cols)

])

def mape(y_true, y_pred):

epsilon = 1e-10

return np.mean(np.abs((y_true - y_pred) / (np.abs(y_true) + epsilon))) * 100

param_grid = {

'regressor__n_estimators': [50, 100],

'regressor__max_depth': [None, 5, 7],

'regressor__min_samples_split': [2, 5],

'regressor__min_samples_leaf': [1, 2],

'regressor__max_features': ['sqrt', 'log2']

}

cv_strategy = KFold(n_splits=10, shuffle=True, random_state=42)

train_metrics = []

test_metrics = []

targets = ['Voc', 'Jsc', 'FF', 'PCE']

model = Pipeline([

('preprocessor', preprocessor),

('regressor', ExtraTreesRegressor(random_state=42))

])

grid_search = GridSearchCV(

estimator=model,

param_grid=param_grid,

cv=cv_strategy,

scoring='neg_mean_squared_error',

n_jobs=-1,

verbose=1

)

grid_search.fit(X_train, y_train[target])

best_model = grid_search.best_estimator_

print(f" optimal parameter：{grid_search.best_params_}")

y_train_pred = best_model.predict(X_train)

y_test_pred = best_model.predict(X_test)

pd.DataFrame({

'real value': y_train[target],

' predicted value ': y_train_pred

}).to_excel(f'{target}_ training set prediction results.xlsx', index=False)

pd.DataFrame({

' real value ': y_test[target],

' predicted value ': y_test_pred

}).to_excel(f'{target}_ test set prediction results.xlsx', index=False)

train_metrics.append({

'Variable': target,

'RMSE': np.sqrt(mean_squared_error(y_train[target], y_train_pred)),

'MAPE': mape(y_train[target], y_train_pred),

'R²': r2_score(y_train[target], y_train_pred)

})

test_metrics.append({

'Variable': target,

'RMSE': np.sqrt(mean_squared_error(y_test[target], y_test_pred)),

'MAPE': mape(y_test[target], y_test_pred),

'R²': r2_score(y_test[target], y_test_pred)

})

**SHAP analysis:**

target = 'PCE'

if target in best_models:

print(f"\n analysis target：{target}")

model = best_models[target]

preprocessed_data = model.named_steps['preprocessor'].transform(X_test)

explainer = shap.TreeExplainer(model.named_steps['regressor'].estimators_[0])

sample_idx = np.random.choice(preprocessed_data.shape[0], 100, replace=False)

shap_values = explainer.shap_values(preprocessed_data[sample_idx])

plt.figure(figsize=(10, 6))

shap.summary_plot(

shap_values,

preprocessed_data[sample_idx],

feature_names=transformed_features,

plot_type='dot',

show=False,

max_display=10

)

plt.title(f"{target} Feature Impact Analysis (SHAP value) ")

plt.tight_layout()

plt.savefig(f'{target}_SHAP analusis.png', dpi=300)

plt.show()

final_report = pd.merge(

pd.DataFrame(train_results),

pd.DataFrame(test_results),

on=' Index ',

suffixes=('_train set', '_test set')

)

**Monte Carlo simulations:**

np.random.seed(42)

n_samples = 500000

monte_carlo_df = pd.DataFrame(columns=[

'Method', 'Substrate-Work Function', 'ETL-HOMO-1', 'ETL-LUMO-1',

'ETL-HOMO-2', 'ETL-LUMO-2', 'MA-ratio', 'FA-ratio', 'Cs-ratio',

'I-ratio', 'Br-ratio', 'HTL-HOMO-1', 'HTL-LUMO-1', 'HTL-HOMO-2',

'HTL-LUMO-2', 'Electrode-Work Function', 'AX evaporation ratio',

'BX2 evaporation ratio', 'Annealing temperature', 'Annealing time',

'pressure', 'thickness', 'Area'

])

for i in range(n_samples):

if (i+1) % 20000 == 0:

print(f"In the generated sample... {i+1}/{n_samples}")

method = 'Sequential vapor deposition'

substrate_wf = 4.85

etl_homo1 = 7.2

etl_lumo1 = 4.2

etl_homo2 = 7.2

etl_lumo2 = 4.2

ma = 0.0

fa = 0.96

cs = 0.04

i_ratio = 1.0

br_ratio = 0.0

htl_homo1 = 5.2

htl_lumo1 = 3.0

htl_homo2 = 5.2

htl_lumo2 = 3.0

electrode_wf = 5.1

ax_ratio = round(np.random.uniform(0.8, 1.2), 2)

bx2_ratio = round(np.random.uniform(2.8, 3.2), 2)

annealing_temp = np.random.randint(120, 180)

annealing_time = np.random.randint(0, 20)

pressure = round(np.random.uniform(0.5, 2.0), 2)

thickness = np.random.randint(300, 400)

area = round(np.random.uniform(0.05, 0.2), 2)

monte_carlo_df.loc[i] = [

method, substrate_wf, etl_homo1, etl_lumo1, etl_homo2, etl_lumo2,

ma, fa, cs, i_ratio, br_ratio, htl_homo1, htl_lumo1, htl_homo2,

htl_lumo2, electrode_wf, ax_ratio, bx2_ratio, annealing_temp,

annealing_time, pressure, thickness, area

]

print("\n The PCE value is being predicted...")

pce_model = best_models['PCE']

monte_carlo_df['PCE'] = pce_model.predict(monte_carlo_df)

print("The Monte Carlo simulation results are being saved...")

monte_carlo_df.to_excel(' Monte Carlo simulation results.xlsx', index=False)

max_index = monte_carlo_df['PCE'].idxmax()

best_combo = monte_carlo_df.loc[max_index]

best_pce = best_combo['PCE']

print(f"\n Find the optimal combination of parameters and predict PCE: {best_pce:.4f}")

best_combo_df = pd.DataFrame([best_combo])

best_combo_df.to_excel(' Optimal parameter combination.xlsx', index=False)

**Prediction of ETree model:**

new_data = pd.read_excel('New data.xlsx')

X_new = new_data.iloc[:, 1:]

method_col = new_data.iloc[:, 0].to_frame()

X_new = pd.concat([method_col, X_new], axis=1)

X_new = X_new[X_train.columns]

predictions = pd.DataFrame()

for target in targets:

predictions[target] = best_models[target].predict(X_new)

full_results = pd.concat([new_data, predictions], axis=1)

full_results.to_excel(' Prediction results.xlsx', index=False)


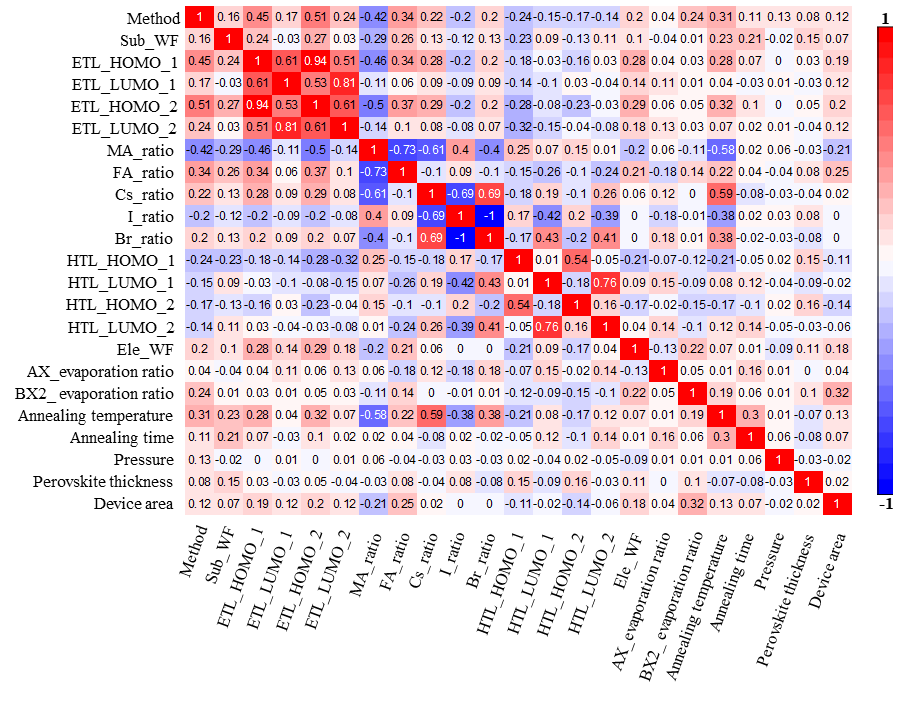


**Figure S1.** Heat maps of Pearson correlation coefficients between the input features.


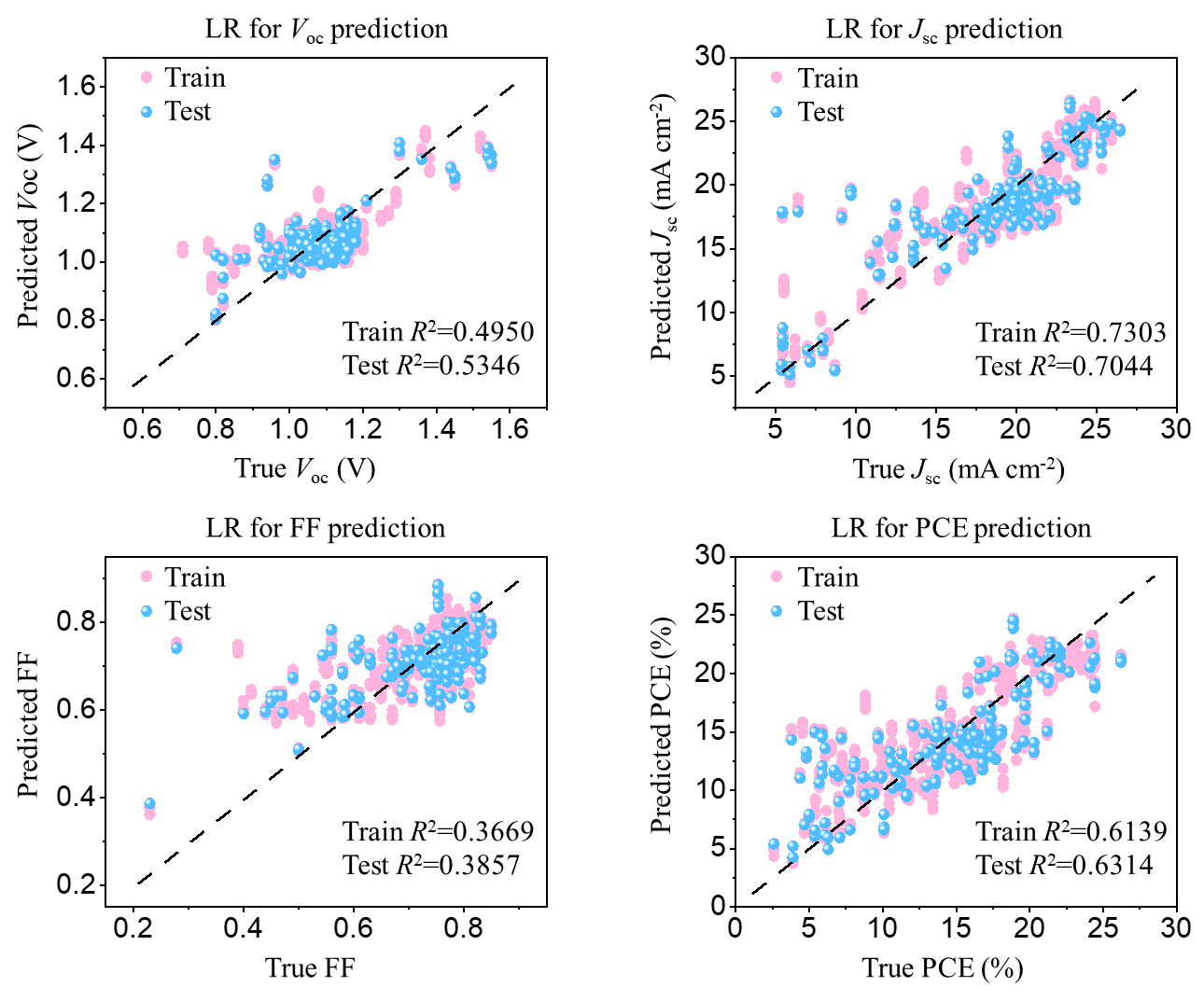


**Figure S2.** The fitting graph of *V*_OC_, *J*_SC_, FF and PCE results by the LR model, where blue represents the training set and pink represents the test set.


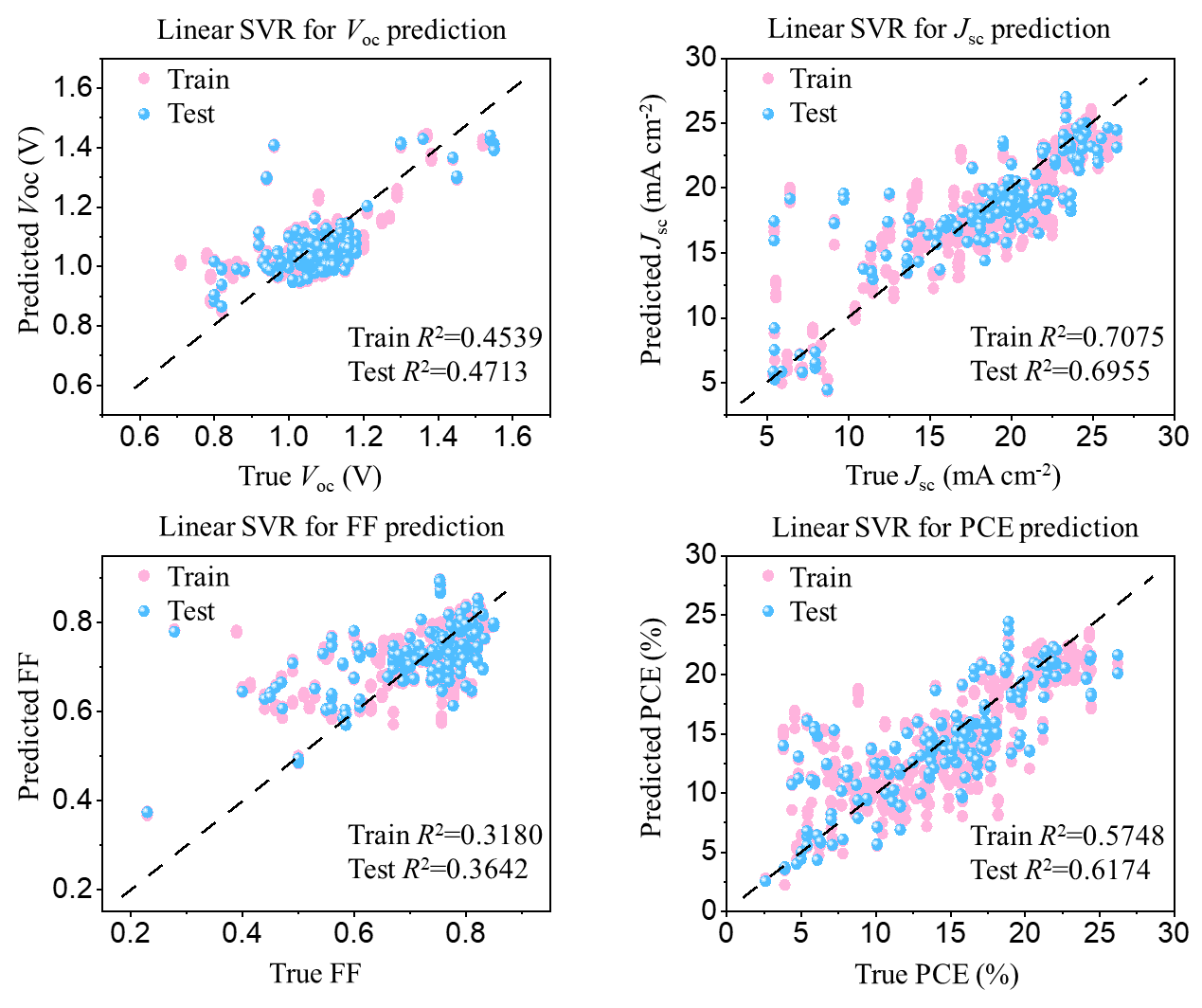


**Figure S3.** The fitting graph of *V*_OC_, *J*_SC_, FF and PCE results by the Linear SVR model, where blue represents the training set and pink represents the test set.


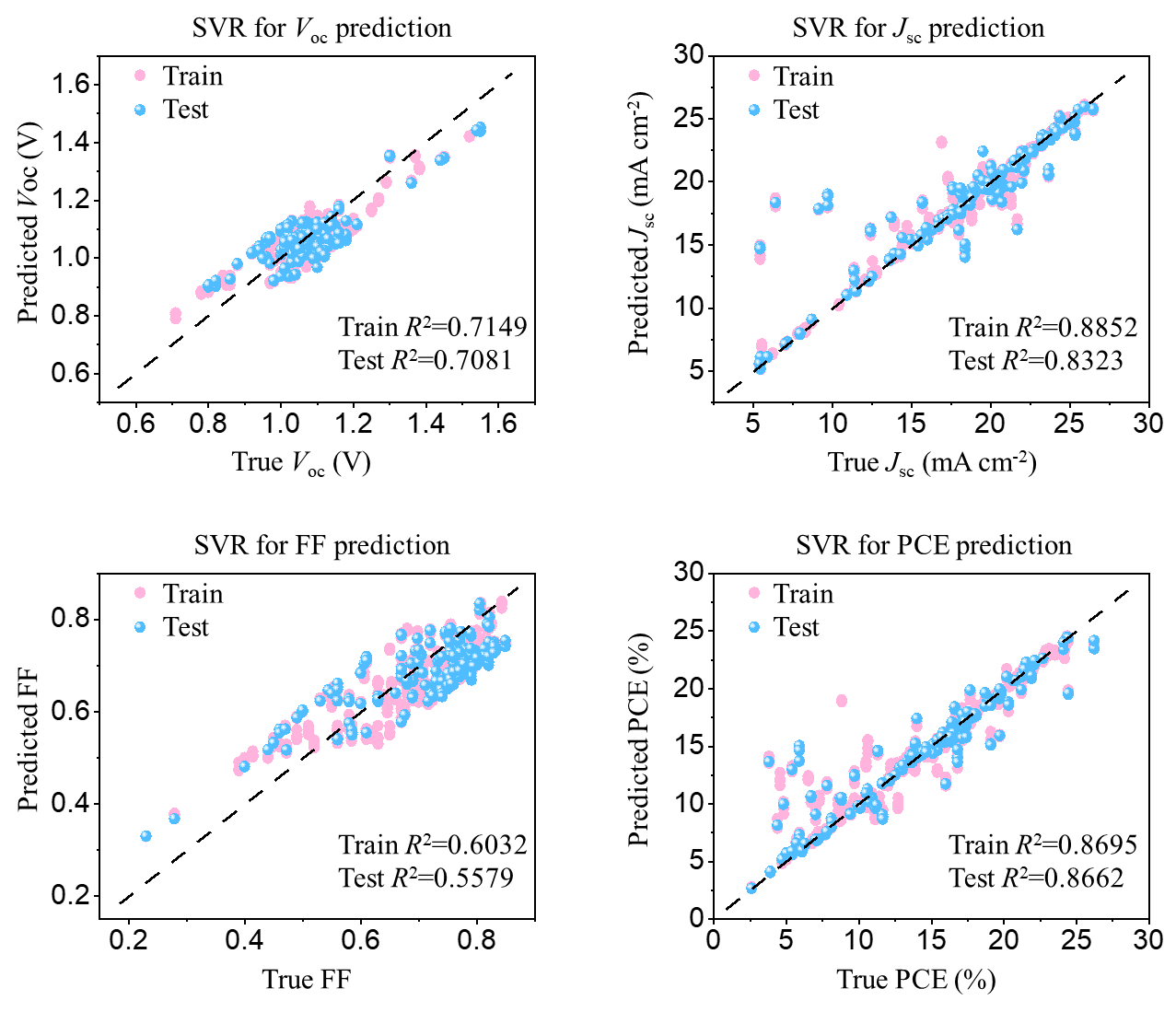


**Figure S4.** The fitting graph of *V*_OC_, *J*_SC_, FF and PCE results by the SVR model, where blue represents the training set and pink represents the test set.


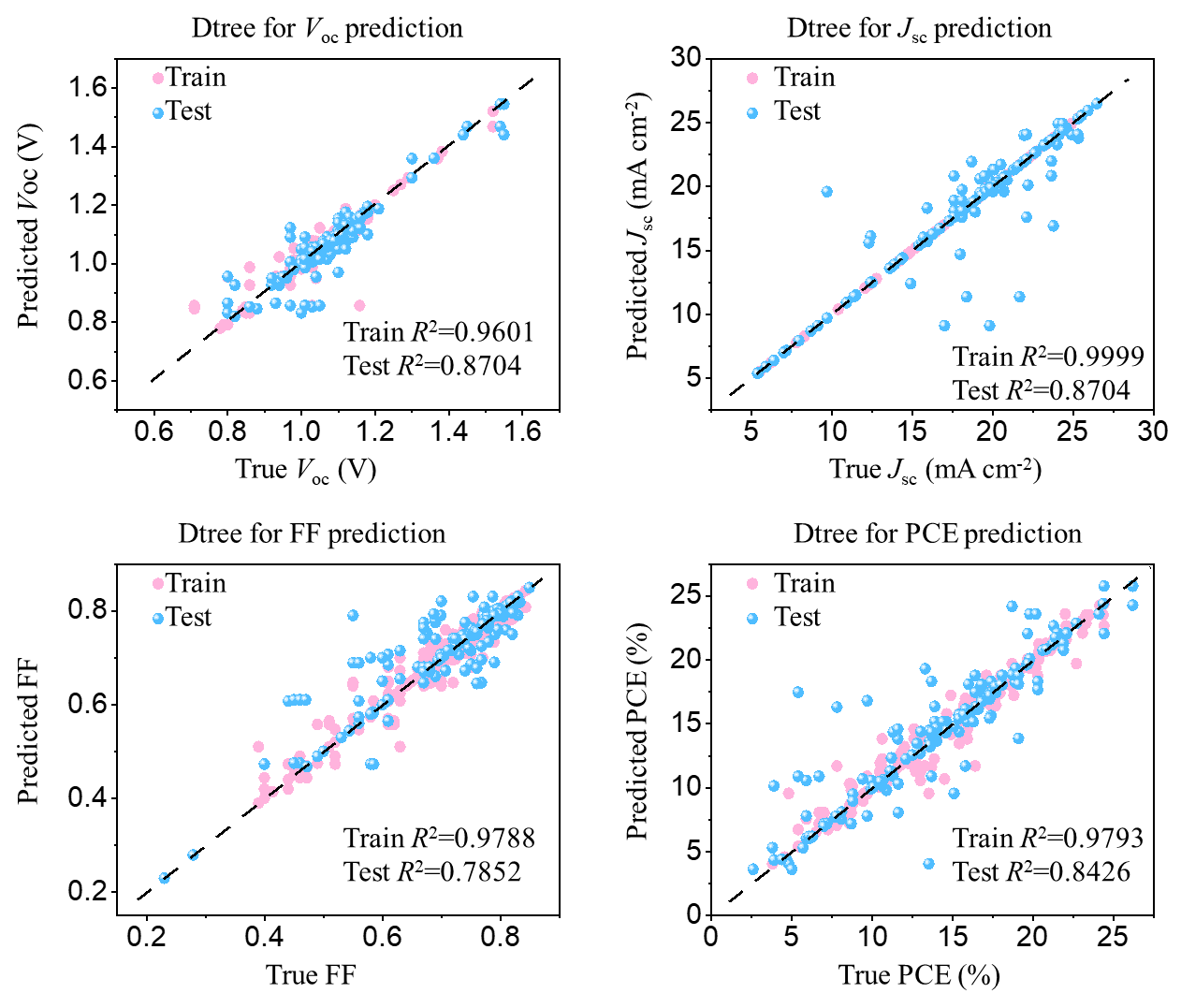


**Figure S5.** The fitting graph of *V*_OC_, *J*_SC_, FF and PCE results by the DTree model, where blue represents the training set and pink represents the test set.


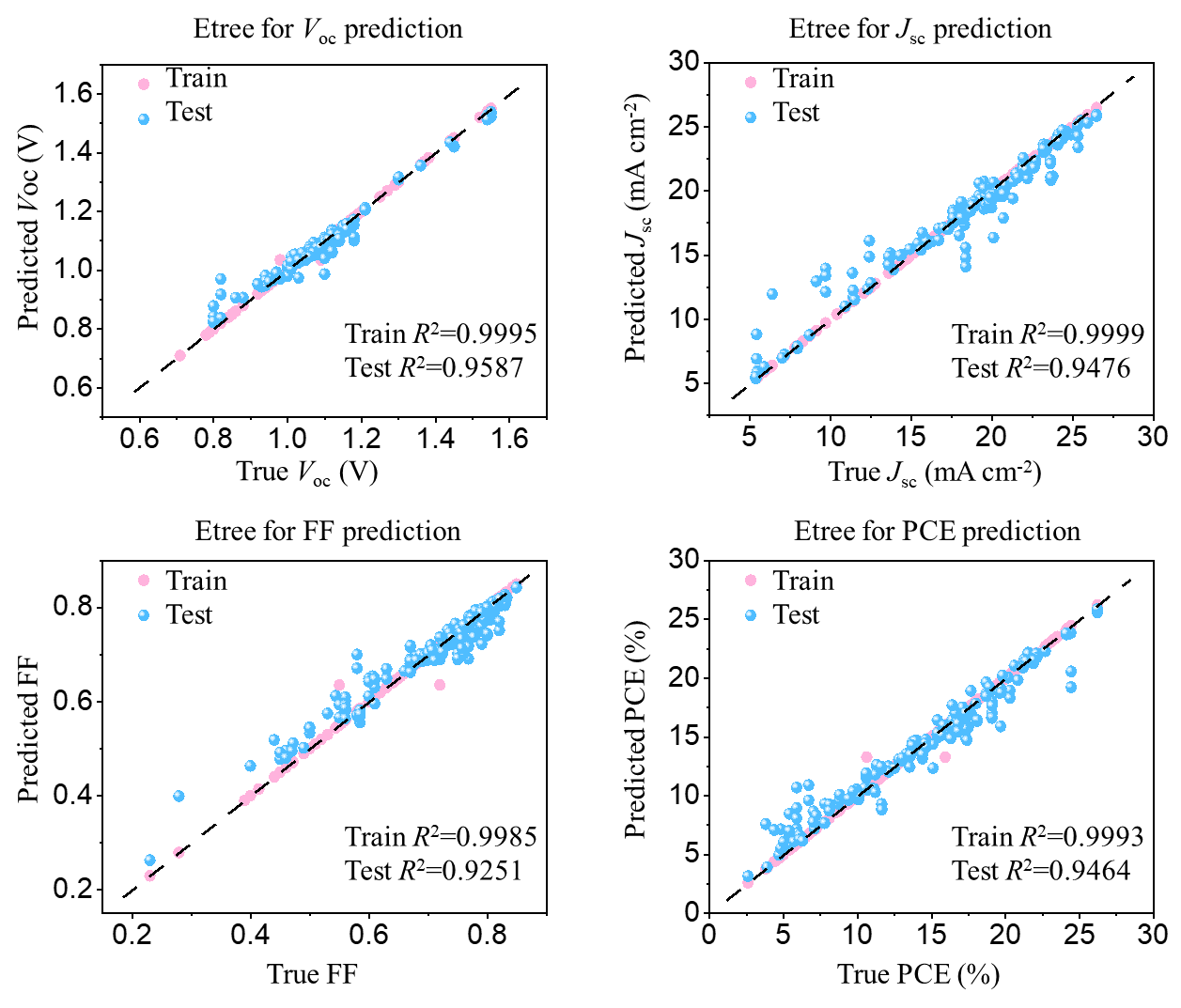


**Figure S6.** The fitting graph of *V*_OC_, *J*_SC_, FF and PCE results by the ETree model, where blue represents the training set and pink represents the test set.


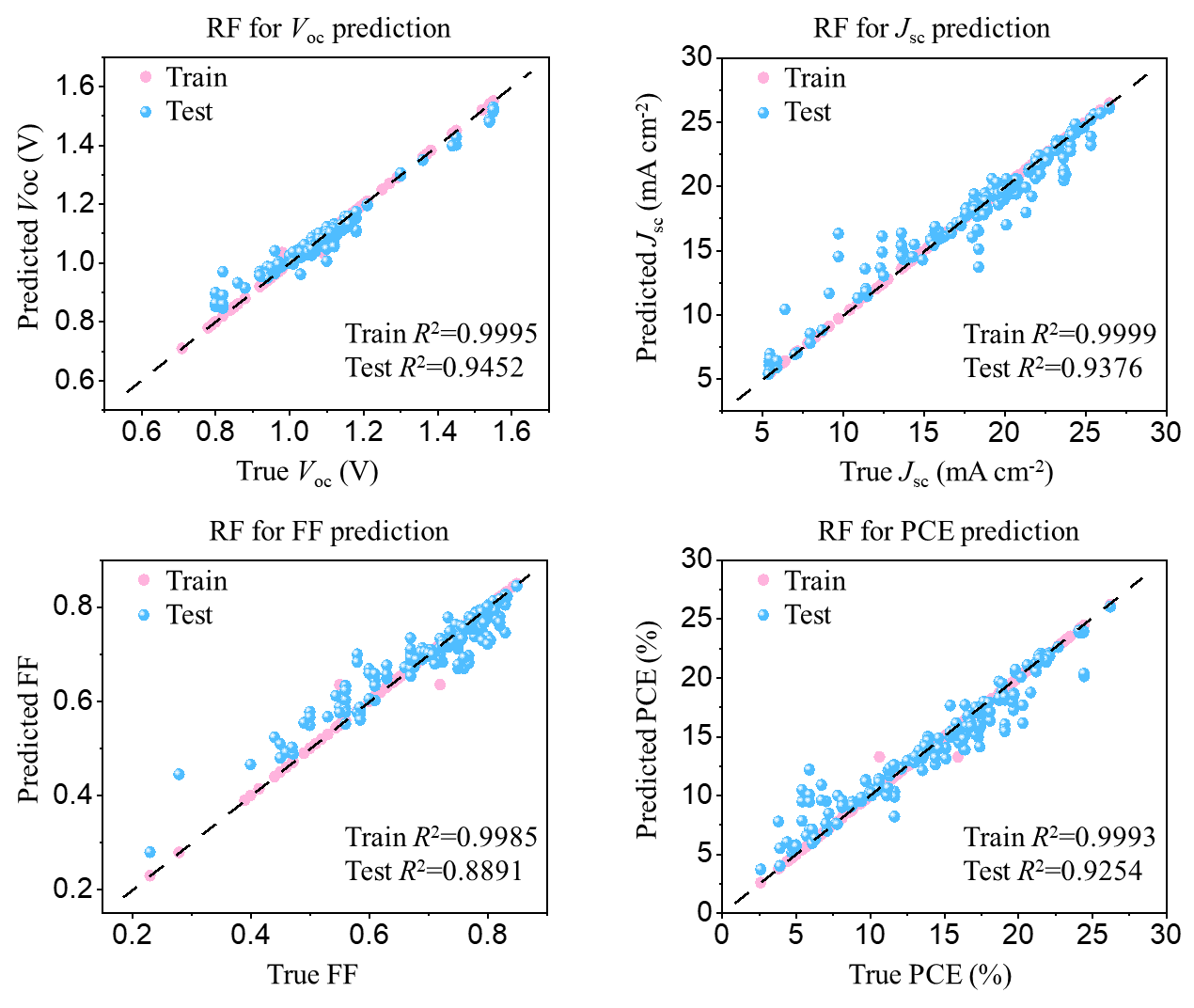


**Figure S7.** The fitting graph of *V*_OC_, *J*_SC_, FF and PCE results by the RF model, where blue represents the training set and pink represents the test set.


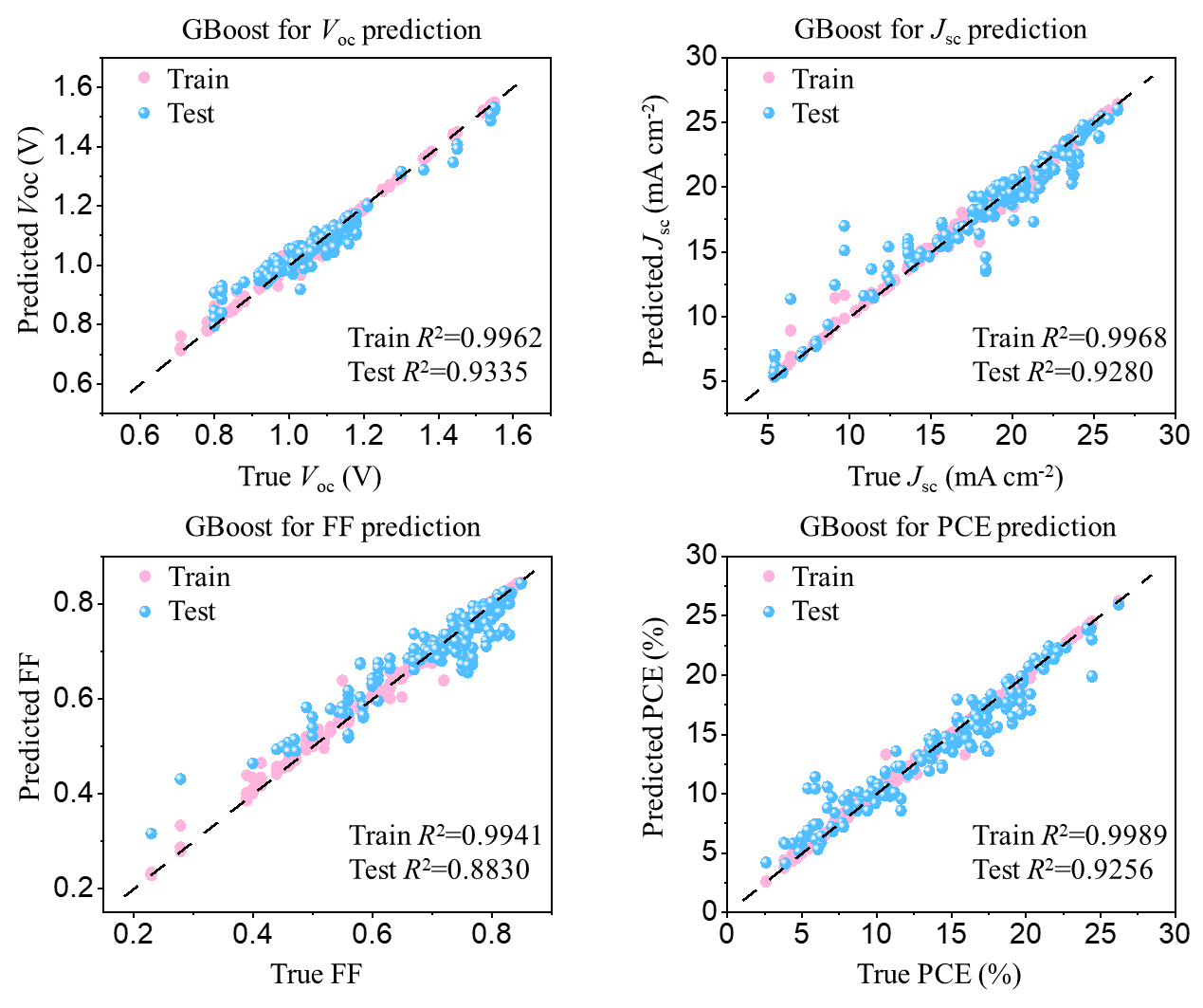


**Figure S8.** The fitting graph of *V*_OC_, *J*_SC_, FF and PCE results by the GBoost model, where blue represents the training set and pink represents the test set.


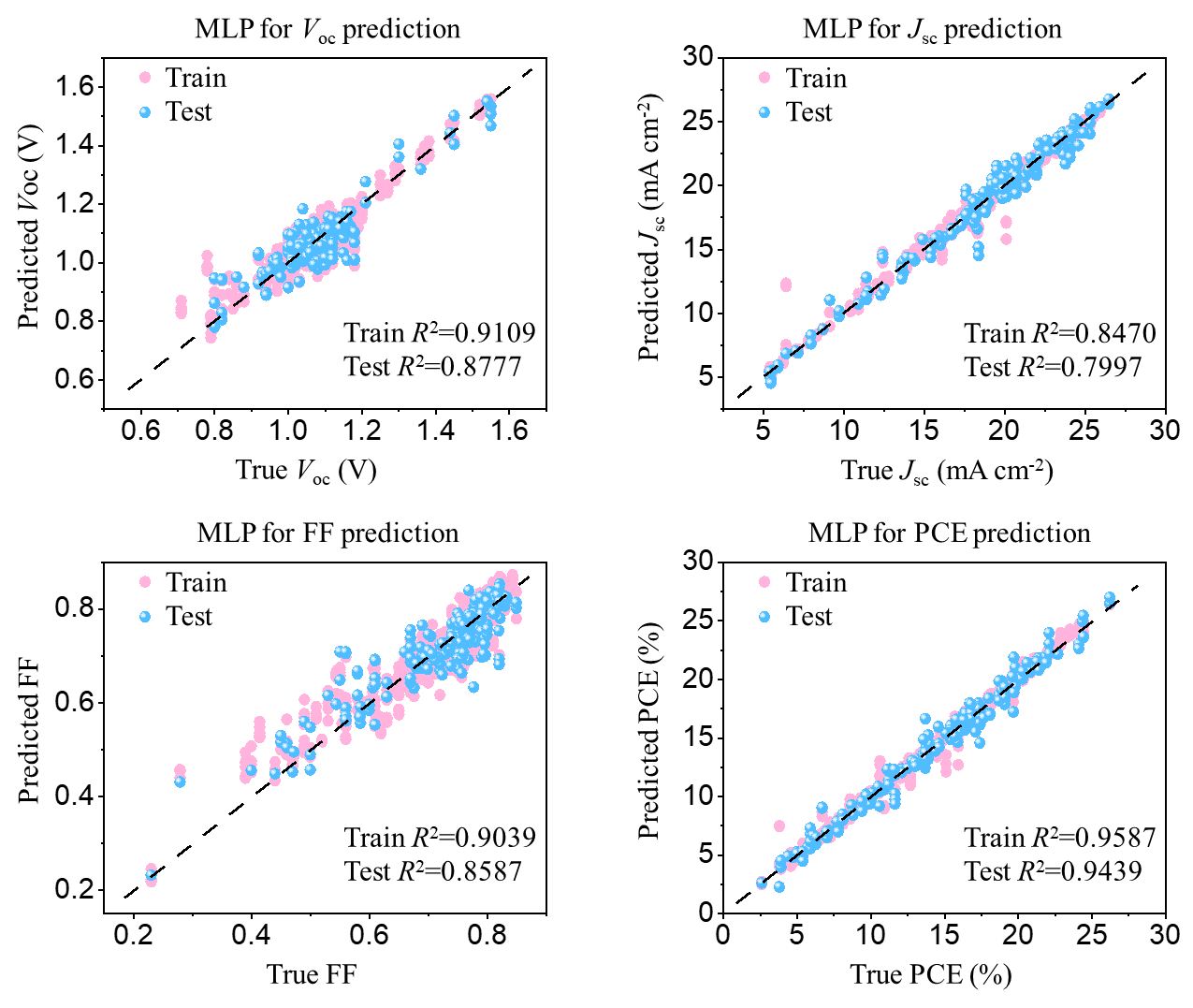


**Figure S9.** The fitting graph of *V*_OC_, *J*_SC_, FF and PCE results by the MLP model, where blue represents the training set and pink represents the test set.


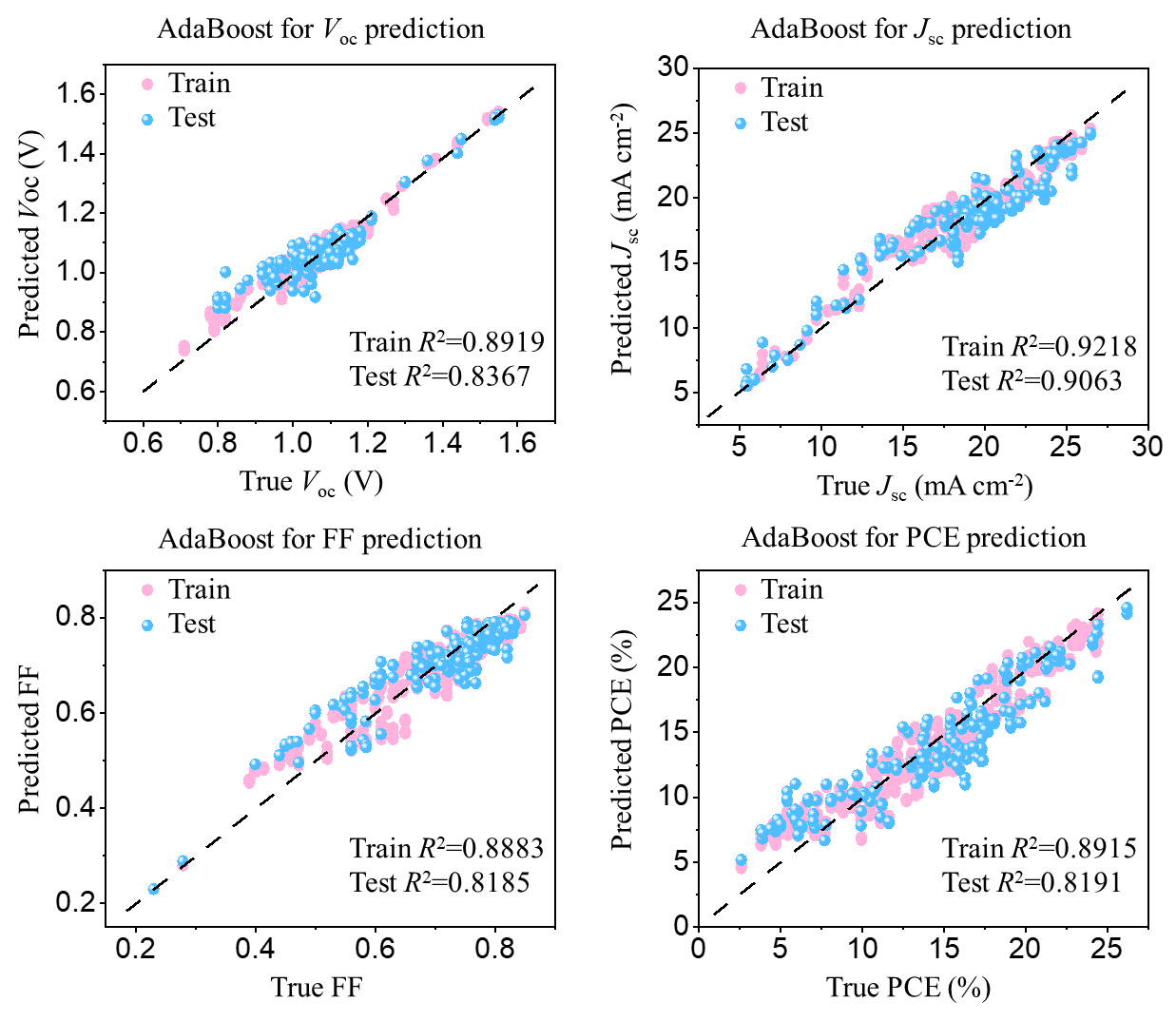


**Figure S10.** The fitting graph of *V*_OC_, *J*_SC_, FF and PCE results by the AdaBoost model, where blue represents the training set and pink represents the test set.


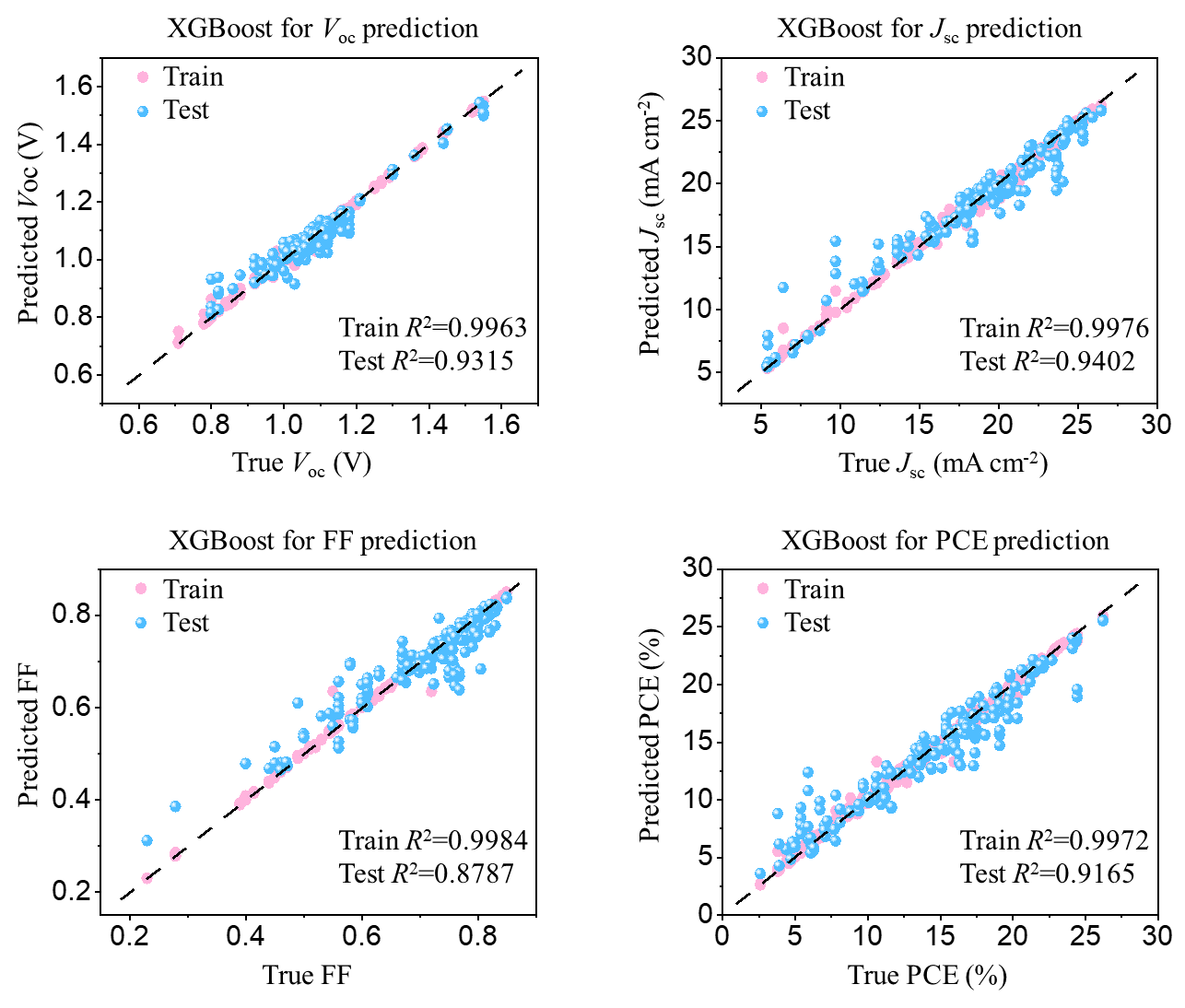


**Figure S11.** The fitting graph of *V*_OC_, *J*_SC_, FF and PCE results by the XGBoost model, where blue represents the training set and pink represents the test set.


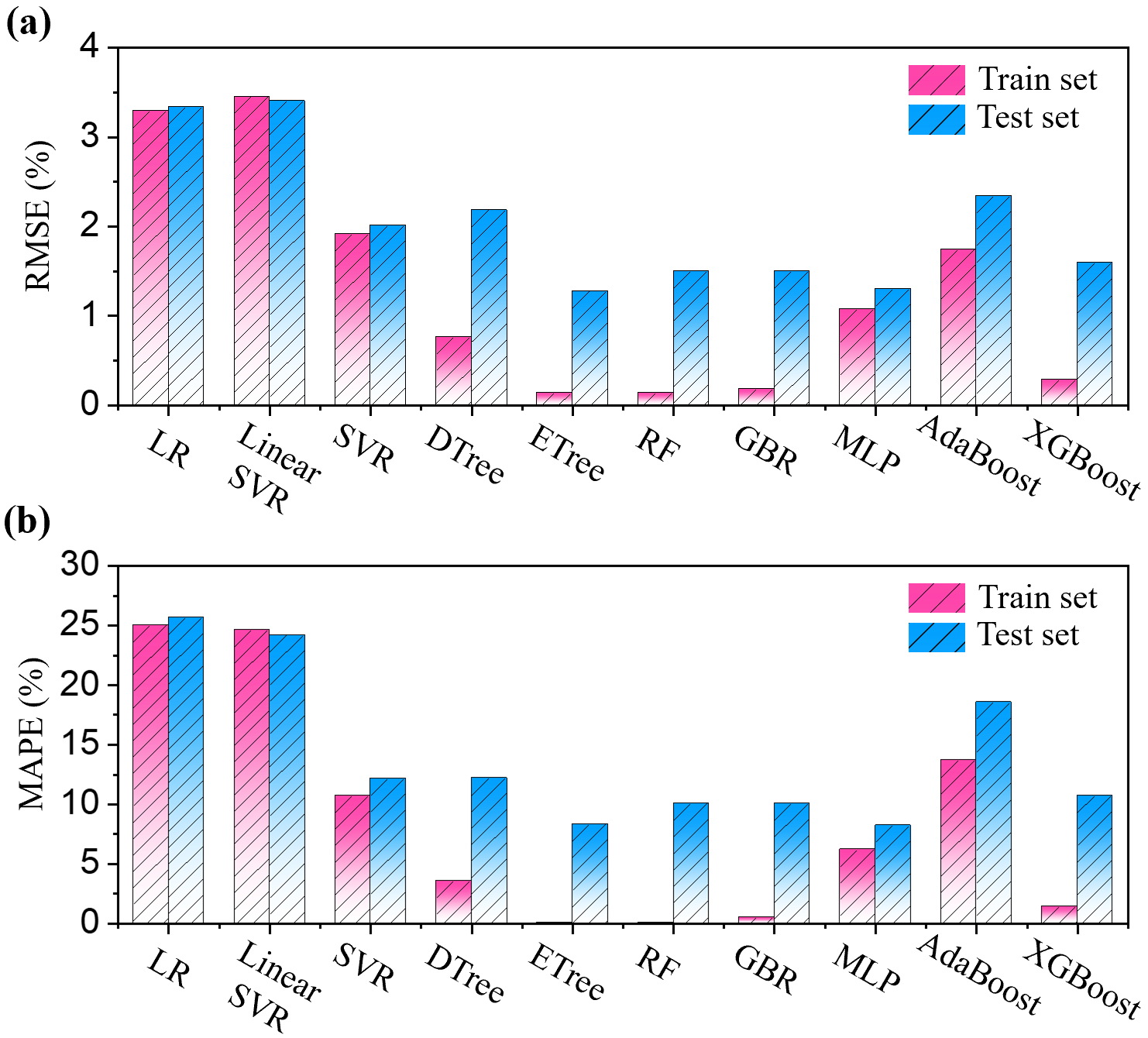


**Figure S12.** RMSE **(a)** and MAPE **(b)** value histograms of predict performance evaluation indicators for 10 ML models.


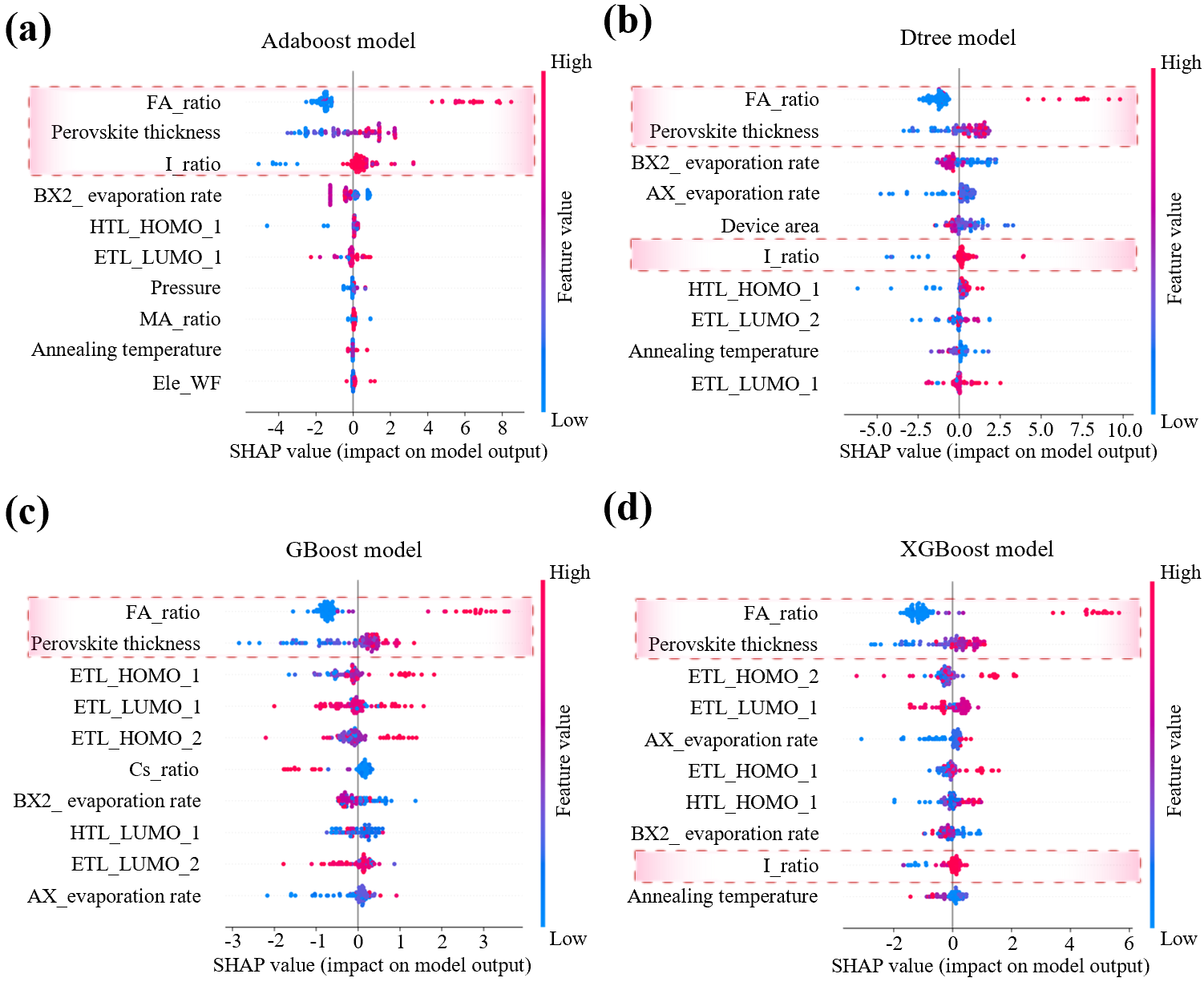


**Figure S13.** The SHAP analysis result graph of all variables contributing to PCE based on AdaBoost model **(a)**, DTree model **(b)**, GBoost model **(c)** and XGBoost model **(d)**.


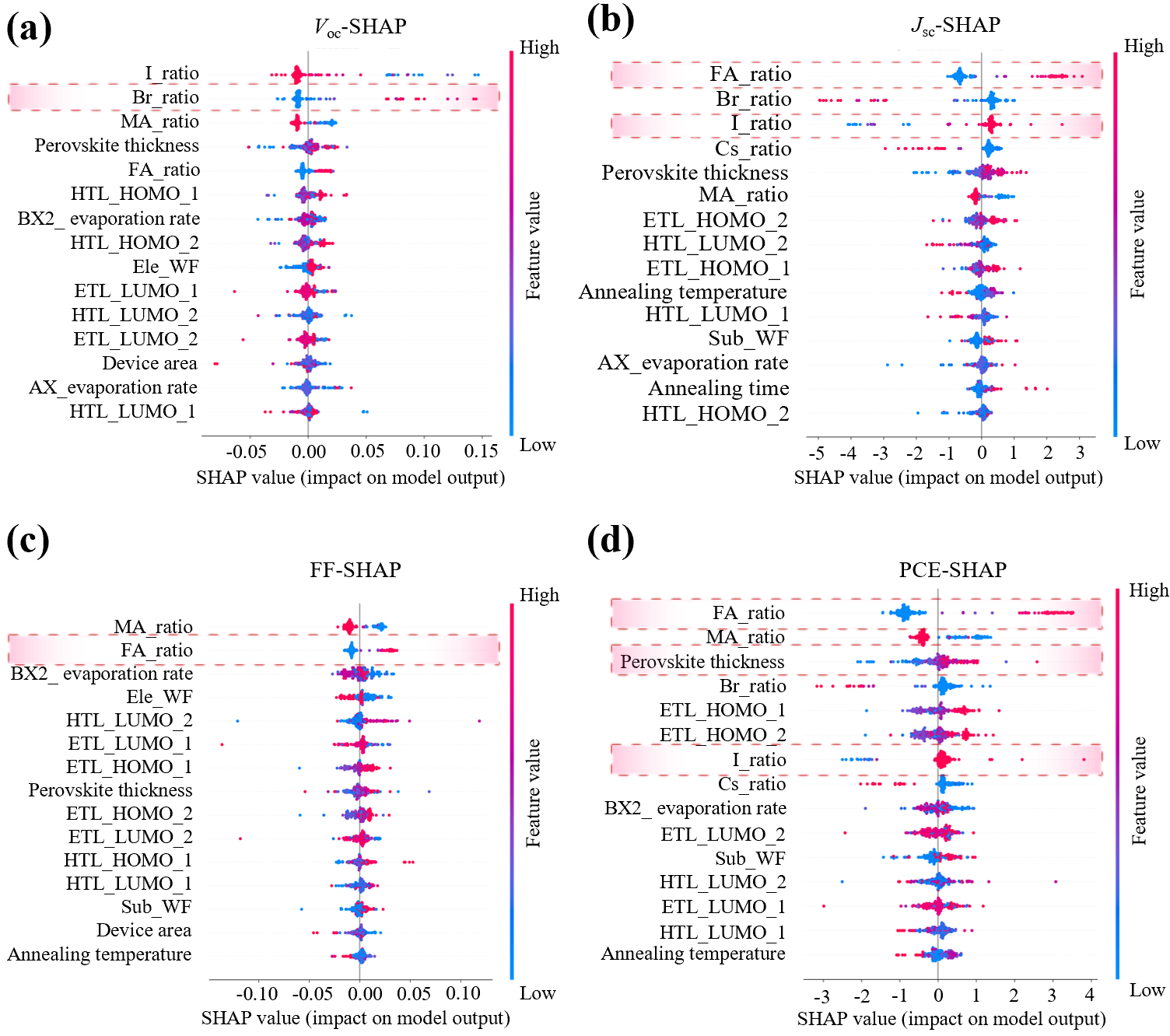


**Figure S14.** The SHAP analysis result graph of all variables respectively contributing to *V*_OC_ **(a)**, *J*_SC_ **(b)**, FF **(c)** and PCE **(d)** based on ETree model.


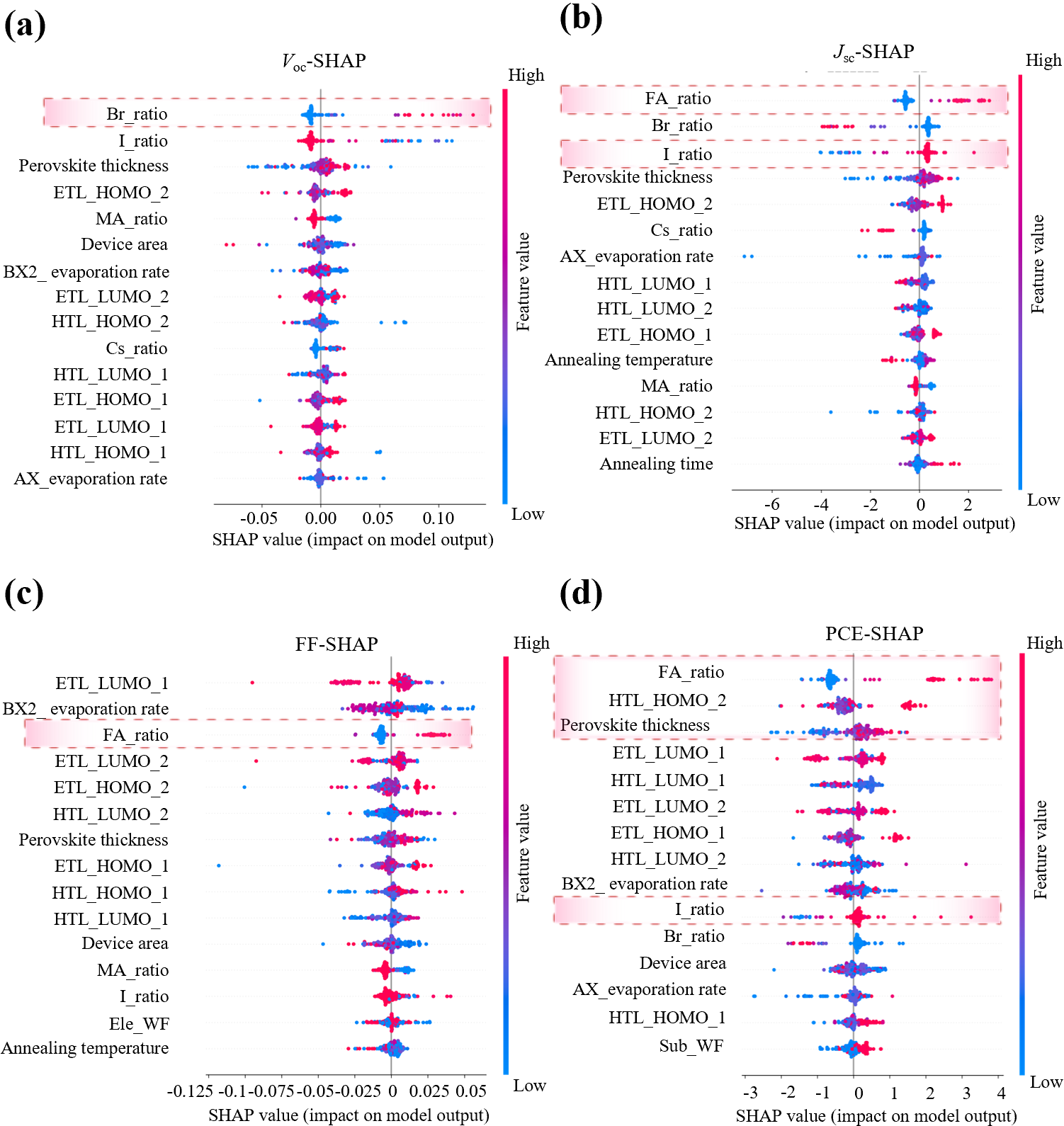


**Figure S15.** The SHAP analysis result graph of all variables respectively contributing to *V*_OC_ **(a)**, *J*_SC_ **(b)**, FF **(c)** and PCE **(d)** based on RF model.


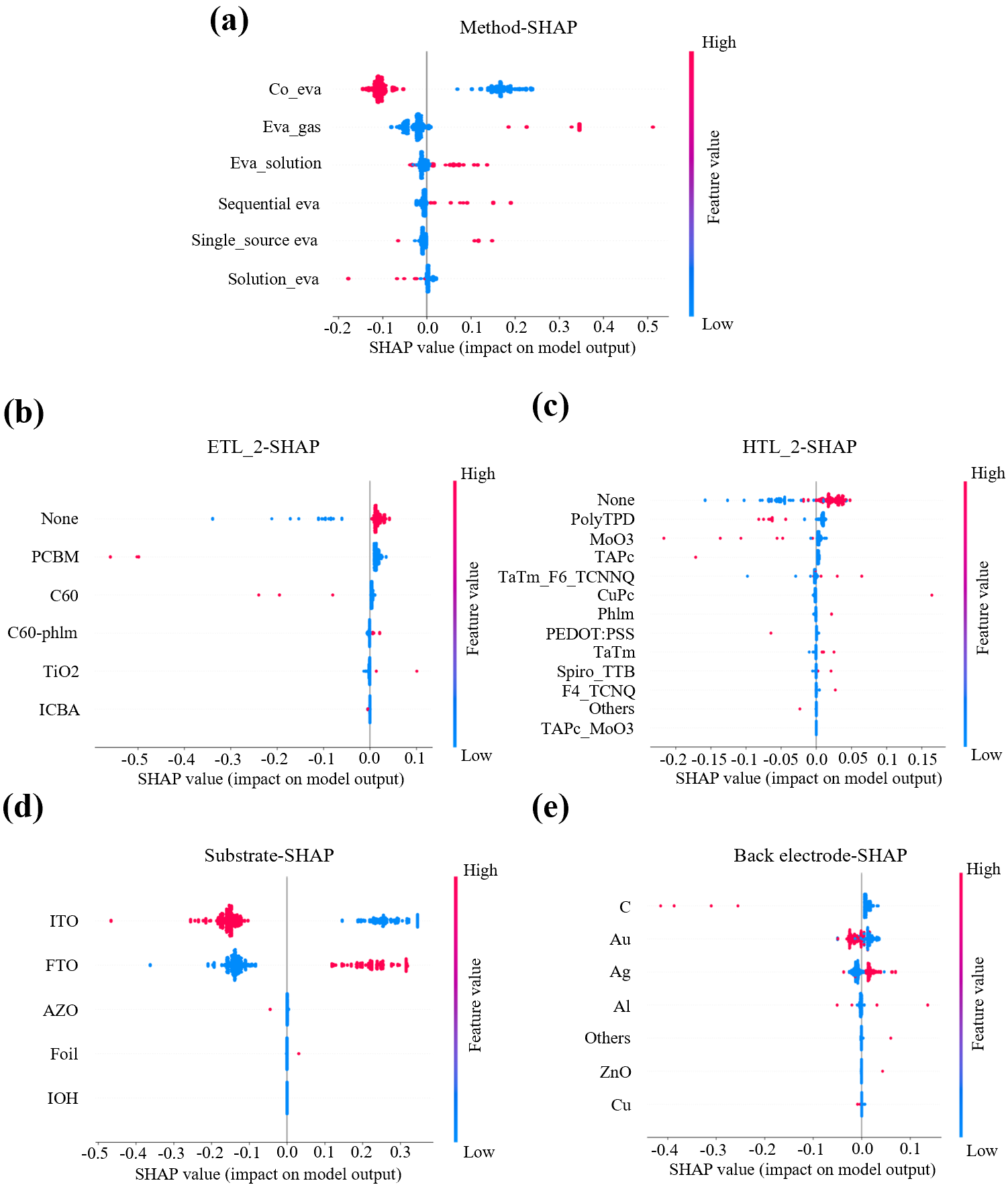


**Figure S16.** The SHAP analysis result graph of Method **(a)**, ETL_2 **(b)**, HTL_2 **(c)**, Substrate **(d)** and Back electrode **(e)** variable respectively contributing to PCE based on ETree model. Note: Co_eva: Co-evaporation; Eva_gas: Evaporation-gas; Eva_solution: Evaporation-solution; Sequential eva: Sequential evaporation; Single_source eva: Single-source evaporation; Solution_eva: Solution-evaporation.


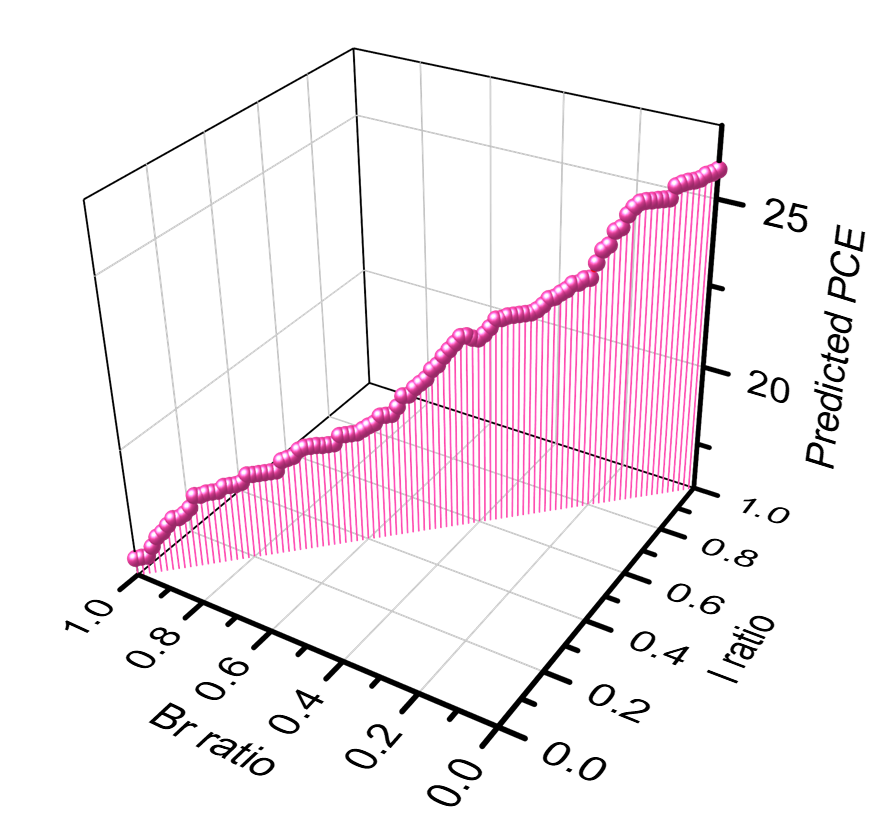


**Figure S17.** The influence of the ratio of I to Br on the predicted PCE.


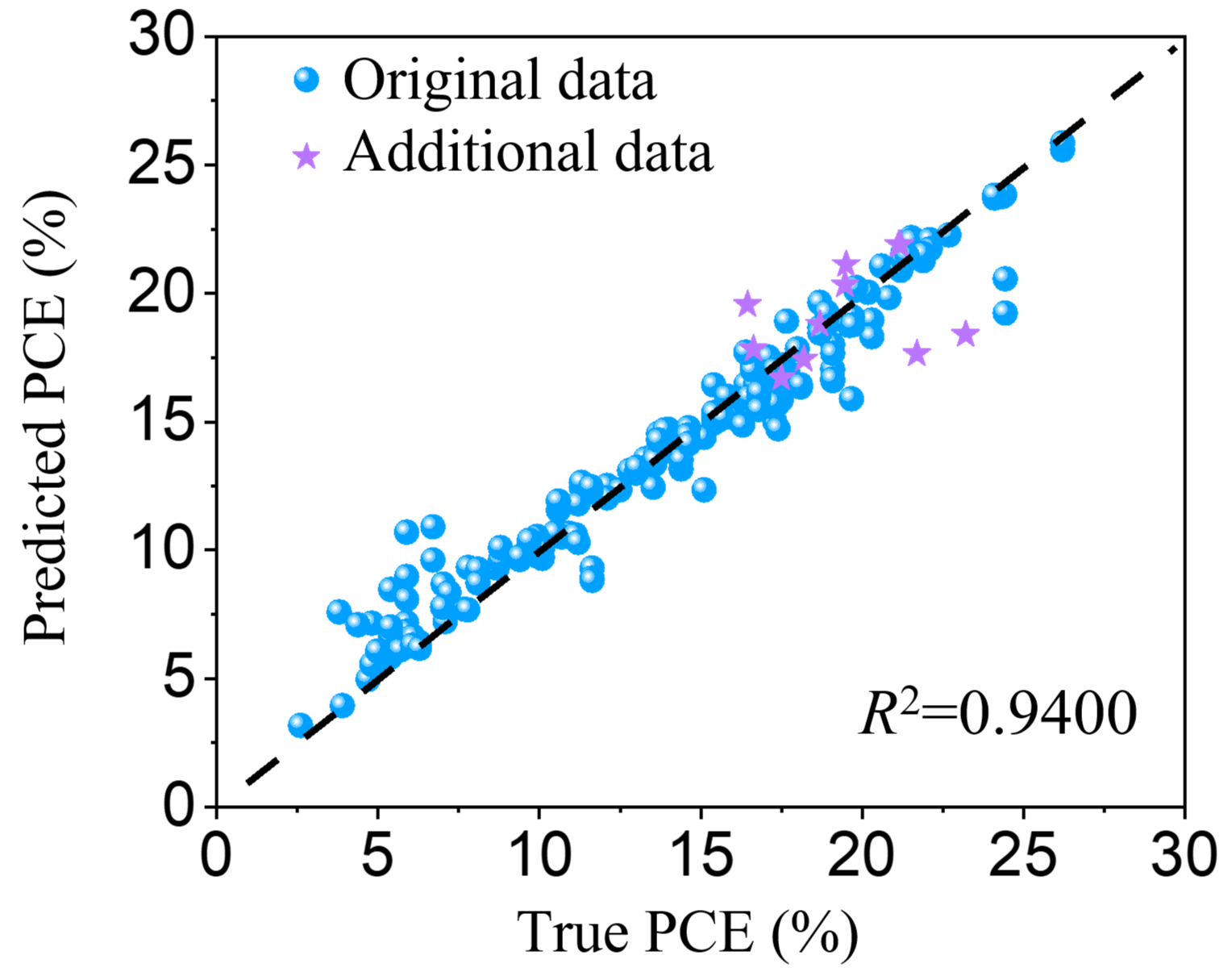


**Figure S18.** The fitting graph of PCE results by the ETree model based on new test set dataset with additional data, where blue ball represents the original data and purple stars represents the test set.


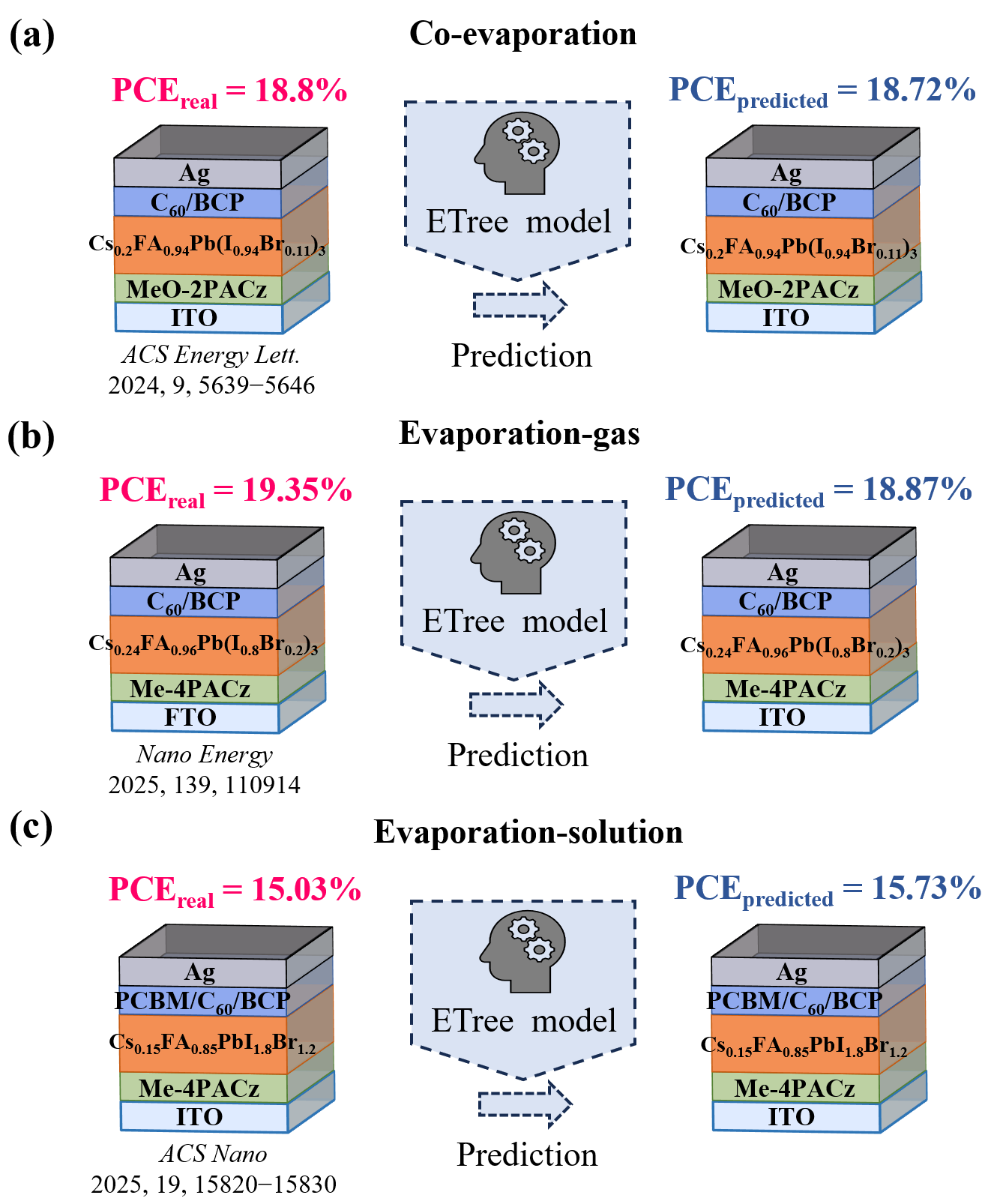


**Figure S19.** PCE prediction based on three different vapor deposition methods and device structures: (a) Co-evaporation, (b) Evaporation-gas, and (c) Evaporation-solution.

**Table S1**. The dataset information and performance evaluation of the reported machine learning models.

| Features | Data volume | ML model | Train set | | | Test set | | | Reference |
| --- | --- | --- | --- | --- | --- | --- | --- | --- | --- |
|  |  |  | *r* | *R*^2^ | RMSE | *r* | *R*^2^ | RMSE |  |
| Microscopic parameters (Pauling’s electronegativity, ionization potential and occupied atomic level, et al.) | ~ 1300 | non-linear regression | / | 0.99 | 0.13 | / | 0.95 | 0.36 | *Sci. Rep.* **2016**, 6, 19375 |
| Microscopic parameters (tolerance factor, octahedron factor and formation energy, et al.) | 3720  (2660) | XGBoost | / | / | / | / | 0.93  (0.97) | 0.25  (0.13) | *J. Mater. Chem. C* **2024**, 12, 15444 |
| Macroscopic parameters (perovskite component, device structure and HTL, et al.) | 269 | Kernel Ridge Regression | 0.68 | / | 3.00 | 0.72 | / | 3.00 | *J. Phys. Chem. C Nanomater. Interfaces* **2022**, 126, 13053 |
| Macroscopic parameters (perovskite composition, fabrication process, and device structure, et al.) | 1072 | XGBoost | 0.91 | / | 0.84 | 0.77 | / | 1.28 | *J. Energy Chem*. **2023**, 77, 200 |
| Macroscopic parameters (perovskite composition, material selection, device structure and manufacturing methods, et al.) | 2079 | CatBoost | 0.94 | 0.88 | 1.89 | 0.87 | 0.76 | 2.69 | *Adv. Funct. Mater.* **2024**, 35, 2410419 |
| Microscopic parameters (Δ_HOMO_, Δ_LUMO_, hole mobility, electron mobility)), Macroscopic parameters (perovskite composition) | 248 | Random forest | / | / | 1.15 | 0.86 | / | 1.58 | *Sol. RRL* **2022**, 6, 2101100 |
| Microscopic parameters (Δ_HOMO_, Δ_LUMO_ and bandgap), Macroscopic parameters (perovskite composition) | 333 | Artificial neural network | / | / | / | 0.80 | / | 3.23 | *Nano Energy* **2022**, 99, 107394 |
| **Microscopic parameters (HOMO, LUMO and work function), Macroscopic parameters (perovskite composition, material selection and manufacturing methods, et al.)** | **202** | **Extra Tree** | **/** | **0.9993** | **0.14** | **/** | **0.9464** | **1.27** | **This work** |

**Table S2.** Samples and data in this work.

| Method | Substrate | ETL_1 | ETL_2 | Perovskite | HTL_1 | HTL_2 | Back electrode | AX evaporation ratio (Å/s) | BX_2_ evaporation ratio (Å/s) | Annealing temperature (℃) | Annealing time (min) | pressure/  mbar (10^-6^) | thickness (nm) | Area (cm²) | *V*_OC_ (V) | *J*_SC_ （mA/cm^2^） | FF (%) | PCE (%) | Reference |
| --- | --- | --- | --- | --- | --- | --- | --- | --- | --- | --- | --- | --- | --- | --- | --- | --- | --- | --- | --- |
| Co-evaporation | FTO | c-TiO_2_ | None | MAPbI_3_ | Spiro-OMeTAD | None | Ag | 5.3 | 1 | 100 | 45 | 10.00 | 330.0 | 0.076 | 1.07 | 21.50 | 67.00 | 15.40 | 10.1038/nature12509 |
| Co-evaporation | ITO | PCBM | None | MAPbI_3_ | PEDOT | polyTPD | Au | / | 0.5 | None | None | 1.00 | 285.0 | 0.09 | 1.05 | 16.12 | 67.00 | 12.00 | 10.1038/NPHOTON.2013.341 |
| Co-evaporation | AZO | PCBM | None | MAPbI_3_ | PEDOT: PSS | None | Au | / | / | None | None | / | 260.0 | 0.12 | 1.04 | 14.30 | 47.00 | 7.00 | 10.1039/C3EE43619E |
| Co-evaporation | FTO | PCBM | None | MAPbI_3-x_Cl_x_ | NiO | None | Ag | 8.5 | 0.95 | 90 | 60 | 7.00 | 375.0 | 0.07 | 0.79 | 14.20 | 65.00 | 7.26 | 10.1021/jz500645n |
| Co-evaporation | ITO | PCBM | None | MAPbI_3_ | PEDOT: PSS | polyTPD | Au | / | 0.5 | 25 | None | 1.00 | 210.0 | 0.09 | 1.04 | 15.40 | 55.00 | 8.80 | 10.1002/aenm.201400345 |
| Co-evaporation | ITO | PCBM | None | MAPbI_3_ | PolyTPD | None | Au | / | / | None | None | / | 250.0 | 0.09 | 1.04 | 16.70 | 61.00 | 10.70 | 10.1002/aenm.201400345 |
| Co-evaporation | ITO | PCBM | None | MAPbI_3_ | PolyTPD | None | Au | / | / | None | None | / | 285.0 | 0.09 | 1.04 | 17.60 | 62.00 | 11.40 | 10.1002/aenm.201400345 |
| Co-evaporation | ITO | PCBM | None | MAPbI_3_ | PolyTPD | None | Au | / | / | None | None | / | 340.0 | 0.09 | 1.02 | 19.30 | 49.00 | 9.70 | 10.1002/aenm.201400345 |
| Co-evaporation | ITO | C_60_ | None | MAPbI_x−3_Cl_x_ | Spiro-MeO-TAD | None | Ag | / | 0.9 | None | None | 0.1 | 195.0 | 0.0644 | 0.80 | 14.40 | 69.00 | 7.80 | 10.1063/1.4889843 |
| Co-evaporation | ITO | C_60_ | None | MAPbI_x−3_Cl_x_ | MeO-TPD | None | Ag | / | / | None | None | 0.1 | 195.0 | 0.0644 | 0.86 | 14.90 | 69.00 | 8.70 | 10.1063/1.4889843 |
| Co-evaporation | ITO | C_60_ | None | MAPbI_x−3_Cl_x_ | Spiro-MeO-TPD | None | Ag | / | / | None | None | 0.1 | 195.0 | 0.0644 | 1.03 | 16.00 | 66.00 | 10.90 | 10.1063/1.4889843 |
| Co-evaporation | ITO | C_60_ | None | MAPbI_x−3_Cl_x_ | Spiro-TTB | None | Ag | / | / | None | None | 0.1 | 195.0 | 0.0644 | 0.97 | 16.10 | 70.00 | 10.90 | 10.1063/1.4889843 |
| Co-evaporation | ITO | C_60_ | None | MAPbI_x−3_Cl_x_ | Spiro-TAD | None | Ag | / | / | None | None | 0.1 | 195.0 | 0.0644 | 0.82 | 12.40 | 58.00 | 6.70 | 10.1063/1.4889843 |
| Co-evaporation | ITO | PCBM | None | MAPbI_3_ | PEDOT | None | Au | / | / | None | None | 1.00 | / | 0.12 | 1.021 | 9.12 | 47.20 | 4.39 | 10.1039/c4ee01389a |
| Co-evaporation | ITO | PCBM | None | MAPbI_3_ | PEDOT | None | Au | / | / | None | None | 1.00 | 100.0 | 0.12 | 1.07 | 11.36 | 58.00 | 7.02 | 10.1039/c4ee01389a |
| Co-evaporation | ITO | PCBM | None | MAPbI_3_ | PEDOT | None | Au | / | / | None | None | 1.00 | 180.0 | 0.12 | 1.08 | 18.38 | 58.50 | 11.63 | 10.1039/c4ee01389a |
| Co-evaporation | ITO | PCBM | None | MAPbI_3_ | PEDOT | None | Au | / | / | None | None | 1.00 | 250.0 | 0.12 | 1.06 | 17.97 | 58.40 | 11.13 | 10.1039/c4ee01389a |
| Co-evaporation | ITO | PCBM | None | MAPbI_3_ | PEDOT | polyTPD | Au | / | / | None | None | 1.00 | 210.0 | 0.06 | 1.04 | 14.70 | 56.00 | 8.60 | 10.1063/1.4890056 |
| Co-evaporation | ITO | PCBM | None | MAPbI_3_ | PEDOT | polyTPD | Au | / | / | None | None | 1.00 | 250.0 | 0.06 | 1.04 | 16.70 | 61.00 | 10.60 | 10.1063/1.4890056 |
| Co-evaporation | ITO | PCBM | None | MAPbI_3_ | PEDOT | polyTPD | Au | / | / | None | None | 1.00 | 285.0 | 0.06 | 1.07 | 18.80 | 63.00 | 12.70 | 10.1063/1.4890056 |
| Co-evaporation | ITO | PCBM | None | MAPbI_3_ | PEDOT | polyTPD | Au |  | / | None | None | 1.00 | 300.0 | 0.06 | 1.10 | 18.10 | 56.00 | 11.20 | 10.1063/1.4890056 |
| Co-evaporation | ITO | PCBM | None | MAPbI_3_ | PEDOT | polyTPD | Au | / | / | None | None | 1.00 | 370.0 | 0.06 | 0.97 | 18.50 | 52.00 | 9.30 | 10.1063/1.4890056 |
| Co-evaporation | ITO | PCBM | None | MAPbI_3_ | PEDOT | polyTPD | Au | / | / | None | None | 1.00 | 390.0 | 0.06 | 1.03 | 18.20 | 46.00 | 8.60 | 10.1063/1.4890056 |
| Co-evaporation | ITO | PCBM | None | MAPbI_3_ | PEDOT | polyTPD | Au | / | / | None | None | 1.00 | 680.0 | 0.06 | 0.94 | 19.50 | 44.00 | 8.10 | 10.1063/1.4890056 |
| Co-evaporation | ITO | PCBM | None | MAPbI_3_ | PEDOT | polyTPD | Au | / | / | None | None | 1.00 | 900.0 | 0.06 | 0.92 | 19.80 | 40.00 | 7.20 | 10.1063/1.4890056 |
| Co-evaporation | ITO | PCBM | None | MAPbI_3_ | PEDOT: PSS | doped-polyTPD | Au | / | / | None | None | 1.00 | 300.0 | 0.06 | 1.03 | 18.00 | 51.00 | 9.60 | 10.1063/1.4890056 |
| Co-evaporation | ITO | PCBM | None | MAPbI_3_ | PEDOT: PSS | doped-polyTPD | Au | / | / | None | None | 1.00 | 900.0 | 0.06 | 0.94 | 19.50 | 65.00 | 12.00 | 10.1063/1.4890056 |
| Co-evaporation | ITO | C_60_ | None | MAPbI_3-x_Cl_x_ | BPhen | None | Al | / | / | 100 | 60 | / | 150.0 | 0.1 | 0.82 | 12.50 | 60.00 | 6.10 | 10.1039/C4TA05819D |
| Co-evaporation | FTO | TiO_2_ | None | MAPbI_3_ | Spiro-OMeTAD | None | Ag | 5.3 | 1 | None | None | 10.00 | 370.0 | 0.2 | 1.05 | 21.90 | 72.00 | 16.50 | 10.1038/NPHOTON.2014.284 |
| Co-evaporation | ITO | C_60_ | None | MAPbI_3_ | NPB | None | Al | / | 0.5 | None | None | **/** | 320.0 | 0.04 | 1.12 | 18.10 | 68.00 | 13.70 | 10.1016/j.orgel.2014.12.002 |
| Co-evaporation | FTO | PCBM | None | MAPbI_3-x_Clₓ | PEDOT: PSS | None | Ag | / | 2 | 100 | 45 | / | 400.0 | 0.12 | 0.97 | 17.30 | 63.00 | 10.50 | 10.1039/c4ra17316c |
| Co-evaporation | / | C_60_ | None | MAPbI_3−x_Cl_x_ | BPhen | None | Al | 0.25 | 0.01 | None | None | 5.00 | / | / | 0.79 | 14.83 | 72.00 | 8.37 | 10.1016/j.orgel.2015.07.028 |
| Co-evaporation | FTO | TiO_2_ | None | MAPbI_3_ | Spiro-OMeTAD | None | Au | / | 0.3 | 100 | 45 | 123.00 | 285.0 | 0.16 | 1.10 | 18.00 | 70.00 | 13.26 | 10.1002/cssc.201500972 |
| Co-evaporation | FTO | C_60_ | None | MAPbI_3_ | CuPc | None | Au | / | 0.75 | None | None | 66.66 | 370.0 | 0.08 | 1.04 | 19.13 | 77.47 | 15.41 | 10.1039/c5ta07829f |
| Co-evaporation | ITO | C_60_ | None | MAPbI_3_ | CuPc | None | Au | / | / | None | None | 66.66 | 370.0 | / | 1.01 | 17.30 | 41.41 | 7.26 | 10.1039/c5ta07829f |
| Co-evaporation | FTO | C_60_ | None | MAPbI_3_ | Spiro-OMeTAD | None | Au | / | 0.75 | None | None | / | 320.0 | 0.08 | 1.10 | 18.90 | 77.70 | 15.70 | 10.1016/j.nanoen.2015.11.008 |
| Co-evaporation | ITO | PCBM | None | MAPbI_3_ | PEDOT: PSS | polyTPD | Ag | / | 0.5 | None | None | / | 320.0 | / | 1.09 | 15.68 | 80.00 | 13.58 | 10.1039/c5ta10574a |
| Co-evaporation | ITO | PCBH | None | MAPbI_3_ | PEDOT: PSS | polyTPD | Ag | / | 0.5 | None | None | / | 320.0 | / | 1.10 | 15.92 | 79.00 | 13.75 | 10.1039/c5ta10574a |
| Co-evaporation | ITO | PCBB | None | MAPbI_3_ | PEDOT: PSS | polyTPD | Ag | / | 0.5 | None | None | / | 320.0 | / | 1.09 | 16.02 | 76.00 | 13.27 | 10.1039/c5ta10574a |
| Co-evaporation | ITO | IPB | None | MAPbI_3_ | PEDOT: PSS | polyTPD | Ag | / | 0.5 | None | None | / | 320.0 | / | 1.10 | 16.28 | 78.00 | 14.02 | 10.1039/c5ta10574a |
| Co-evaporation | ITO | IPH | None | MAPbI_3_ | PEDOT: PSS | polyTPD | Ag | / | 0.5 | None | None | / | 320.0 | / | 1.11 | 16.70 | 79.00 | 14.64 | 10.1039/c5ta10574a |
| Co-evaporation | FTO | c-TiO₂ | None | CsPbIBr_2_ | None | None | Au | 0.21 | 0.2 | 250 | 20 | 1.00 | 190.0 | 0.159 | 0.96 | 8.70 | 56.00 | 4.70 | 10.1002/aenm.201502202 |
| Co-evaporation | ITO | C_60_ | None | MAPbI_3_ | NPB | None | Al | / | 0.5 | None | None | 67.83 | 320.0 | 0.04 | 1.05 | 19.73 | 69.00 | 14.50 | 10.1039/c6ta00168h |
| Co-evaporation | ITO | PCBM | None | MAPbI_3_ | PEDOT: PSS | polyTPD | Ag | / | 0.5 | None | None | 1.00 | 500.0 | / | 1.109 | 19.90 | 68.70 | 15.10 | 10.1039/c6ee00462h |
| Co-evaporation | ITO | C_60_ | None | MAPbI_3_ | PEDOT: PSS | polyTPD | Ag | / | / | None | None | 1.00 | 500.0 | / | 1.11 | 20.50 | 64.60 | 14.70 | 10.1039/c6ee00462h |
| Co-evaporation | ITO | C_60_ | None | MASnI_3_ | PSS | None | Ag | / | 1 | None | None | 106.00 | 200.0 | 0.04 | 0.49 | 12.10 | 36.60 | 1.70 | 10.1039/c6ra19476a |
| Co-evaporation | ITO | C_60_ | C_60_: PhIm | MAPbI_3_ | TaTm | TaTm: F6-TCNNQ | Ag | / | / | None | None | 1.00 | 500.0 | 0.01 | 1.08 | 20.02 | 73.30 | 15.40 | 10.1039/c6ee02100j |
| Co-evaporation | ITO | C_60_ | C_60_: PhIm | MAPbI_3_ | TaTm | TaTm: F6-TCNNQ | Au | / | / | None | None | 1.00 | 500.0 | 0.01 | 1.12 | 20.28 | 83.00 | 20.30 | 10.1039/c6ee02100j |
| Co-evaporation | ITO | TiOx | None | CsPbI_3_ | P3HT | None | Au | 3 | 3 | None | None | 6.67 | 300.0 | 0.45 | 1.06 | 13.90 | 71.60 | 10.50 | 10.1021/acs.jpclett.6b02594 |
| Co-evaporation | ITO | C_60_: PhIm | None | MAPbI_3_ | TaTm: F6-TCNNQ | None | Au | / | / | None | None | 1.00 | 500.0 | 0.06 | 1.06 | 20.64 | 78.80 | 17.30 | 10.1002/aenm.201602121 |
| Co-evaporation | FTO | TiO_2_ | None | MAPbI_3_ | Spiro-OMeTAD | None | Ag | 0.4 | 0.4 | None | None | 1.00 | 300.0 | 0.0919 | 1.04 | 20.20 | 75.00 | 15.80 | 10.1002/aelm.201600470 |
| Co-evaporation | FTO | C_60_ | None | MAPbI_3_ | Spiro-OMeTAD | None | Ag | 0.4 | 0.4 | None | None | 1.00 | 300.0 | 0.0919 | 1.07 | 21.30 | 68.00 | 15.40 | 10.1002/aelm.201600470 |
| Co-evaporation | FTO | PCBM | None | MAPbI_3_ | Spiro-OMeTAD | None | Ag | 0.4 | 0.4 | None | None | 1.00 | 300.0 | 0.0919 | 1.10 | 20.60 | 61.00 | 13.60 | 10.1002/aelm.201600470 |
| Co-evaporation | ITO | C_60_ | None | MAPbI_3-x_Cl_x_ | Spiro-TTB | None | Ag | 1.3 | 0.65 | None | None | 1.00 | 380.0 | 0.064 | 1.00 | 17.60 | 67.00 | 11.30 | 10.1002/ente.201700002 |
| Co-evaporation | ITO | C_60_ | None | CsPbI_3_ | TAPC: MoO3 | TAPC | Ag | 0.875 | 0.91 | 325 | 1 | 2.67 | 300.0 | 0.051 | 0.98 | 17.30 | 56.00 | 9.40 | 10.1002/adma.201605290 |
| Co-evaporation | ITO | C_60_ | None | CsPbI_2_Br | TAPC: MoO3 | TAPC | Ag | 0.5 | 0.78 | 260 | / | 2.67 | 400.0 | 0.051 | 1.13 | 15.20 | 68.00 | 11.80 | 10.1002/adma.201605290 |
| Co-evaporation | ITO | PCBM | None | CsPbBr_3_ | PEDOT: PSS | None | Ag | 5 | 5 | 130 | 15 | 6.67 | 300.0 | 0.45 | 0.94 | 5.90 | 70.00 | 3.9±0.5 | 10.1016/j.solmat.2017.06.059 |
| Co-evaporation | FTO | TiO₂ | None | CsPbI_3_ | P3HT | None | Ag | 0.3 | 0.5 | 350 | 1 | 4.00 | 200.0 | 0.04 | 0.79 | 12.06 | 72.00 | 6.79 | 10.1021/acsomega.7b00814 |
| Co-evaporation | FTO | TiO₂ | None | CsPbI_2_Br | P3HT | None | Au | 0.14 | 0.22 | 300 | 10 | 1.00 | 230.0 | 0.159 | 1.04 | 11.50 | 67.00 | 7.70 | 10.1021/acs.jpcc.7b06268 |
| Co-evaporation | FTO | C_60_ | None | FAPbI_3_ | Spiro-OMeTAD | None | Ag | / | / | 170 | 1 | 1.00 | 300.0 | 0.0919 | 1.01 | 22.10 | 69.40 | 15.80 | 10.1021/acsenergylett.7b00967 |
| Co-evaporation | ITO | C_60_ | C_60_: PhIm | MAPbI_3_ | F6-TCNNQ | PhIm | Au | / | / | 90 | 15 | 1.00 | 450.0 | / | 1.10 | 19.60 | 81.30 | 17.50 | 10.1021/acsenergylett.7b01217 |
| Co-evaporation | ITO | C_60_ | C_60_: PhIm | MAPb(Br_0.2_I_0.8_)_3_ | F6-TCNNQ | PhIm | Au | / | / | 90 | 15 | 1.00 | 450.0 | / | 1.12 | 17.30 | 82.30 | 15.90 | 10.1021/acsenergylett.7b01217 |
| Co-evaporation | ITO | C_60_ | C_60_: PhIm | MAPb(Br_0.5_I_0.5_)_3_ | F6-TCNNQ | PhIm | Au | / | / | 90 | 15 | 1.00 | 450.0 | / | 1.21 | 11.40 | 76.90 | 10.60 | 10.1021/acsenergylett.7b01217 |
| Co-evaporation | ITO | TiO₂ | None | MAPbI_3_ | TaTm | TaTm: F6-TCNNQ | Au | / | / | None | None | 1.00 | 970.0 | 0.0625 | 1.16 | 21.91 | 82.09 | 20.83 | 10.1021/acs.jpclett.7b03361 |
| Co-evaporation | ITO | C_60_ | C_60_: PhIm | Cs_0.5_FA_0.5_Pb(I_0.83_Br_0.17_)_3_ | TaTm: F6-TCNNQ | None | Au | 0.68 | 1 | None | None | 40.00 | 310.0 | / | 0.92 | 18.70 | 56.00 | 9.70 | 10.1002/aenm.201703506 |
| Co-evaporation | ITO | C_60_ | C_60_: PhIm | Cs_0.5_FA_0.4_MA_0.1_Pb(I_0.83_Br_0.17_)_3_ | TaTm: F6-TCNNQ | None | Au | / | / | None | None | 40.00 | 340.0 | / | 1.14 | 16.90 | 81.00 | 15.60 | 10.1002/aenm.201703506 |
| Co-evaporation | ITO | TiO₂ | C₆₀ | MAPbI_3_ | TaTm | MoO3 | Au | / | / | None | None | 1.00 | 500.0 | 0.0653 | 1.10 | 19.70 | 77.10 | 16.70 | 10.1021/acsenergylett.8b00193 |
| Co-evaporation | ITO | ZnO | None | CsPbBr_3_ | spiro-OMeTAD | None | Au | 5 | 5 | 180 | 15 | 66.50 | 450.0 | 0.1 | 1.44 | 7.01 | 77.11 | 7.78 | 10.1002/adma.201800855 |
| Co-evaporation | ITO | C_60_ | C_60_: PhIm | MAPbI_3_ | TaTm | TaTm: F6-TCNNQ | Ag | 0.8 | 0.5 | None | None | 1.00 | 600.0 | / | 1.06 | 20.10 | 71.10 | 15.10 | 10.1021/acs.jpclett.8b00964 |
| Co-evaporation | ITO | C_60_ | C_60_: PhIm | MAPbI_3_ | TaTm | TaTm: F6-TCNNQ | Ag | 0.8 | 0.4 | None | None | 1.00 | 600.0 | / | 1.03 | 18.30 | 72.00 | 13.53 | 10.1021/acs.jpclett.8b00964 |
| Co-evaporation | ITO | C_60_ | C_60_: PhIm | MAPbI_3_ | TaTm | TaTm: F6-TCNNQ | Ag | 0.8 | 1.2 | None | None | 1.00 | 600.0 | / | 1.07 | 6.40 | 54.40 | 3.80 | 10.1021/acs.jpclett.8b00964 |
| Co-evaporation | ITO | C_60_: PhIm | None | MAPbI_3_ | TaTm | TaTm: F6-TCNNQ | Ag | / | / | None | None | 1.00 | 500.0 | 0.01 | 1.07 | 19.60 | 75.00 | 15.70 | 10.1039/c8se00218e |
| Co-evaporation | ITO | C_60_: PhIm | None | MAPbI_3_ | TaTm | None | Ag | / | / | None | None | 1.00 | 500.0 | 0.01 | 1.03 | 18.80 | 27.90 | 5.40 | 10.1039/c8se00218e |
| Co-evaporation | ITO | C_60_: PhIm | None | MAPbI_3_ | TaTm | TaTm: F6-TCNNQ | Ag | / | / | None | None | 1.00 | 500.0 | 0.01 | 1.06 | 20.70 | 79.10 | 17.40 | 10.1039/c8se00218e |
| Co-evaporation | ITO | C_60_ | None | MAPbI_3_ | TaTm | TaTm: F6-TCNNQ | Ag | / | / | None | None | 1.00 | 500.0 | 0.01 | 1.06 | 19.20 | 80.50 | 16.40 | 10.1039/c8se00218e |
| Co-evaporation | ITO | C_60_ | None | MAPbI_3_ | TaTm | TaTm: F6-TCNNQ | Ag | / | / | None | None | 1.00 | 500.0 | 0.01 | 1.07 | 20.60 | 80.70 | 17.70 | 10.1039/c8se00218e |
| Co-evaporation | FTO | TiO₂ | PCBM | MAPbI_3_ | Spiro-OMeTAD | None | Au | 0.5 | / | 100 | 30 | 10.00 | 380.0 | 0.16 | 1.01 | 21.30 | 75.00 | 17.10 | 10.1021/acsami.8b07999 |
| Co-evaporation | FTO | TiO₂ | None | MAPbI_3_ | Spiro-OMeTAD | None | Au | 0.5 | / | 100 | 30 | 10.00 | 380.0 | 0.16 | 0.97 | 9.70 | 63.00 | 5.90 | 10.1021/acsami.8b07999 |
| Co-evaporation | FTO | C_60_ | None | MAPbI_3_ | spiro-OMeTAD | None | Ag | / | / | 100 | 60 | 1.00 | / | 0.0919 | 1.07 | 22.30 | 68.00 | 15.70 | 10.1039/c8me00031j |
| Co-evaporation | FTO | PTCBI | None | MAPbI_3_ | Spiro-OMeTAD | None | Ag | / | / | 100 | 60 | 1.00 | / | 0.0919 | 1.04 | 22.20 | 61.00 | 13.70 | 10.1039/c8me00031j |
| Co-evaporation | FTO | (RhCp*Cp)2/PTCBI | None | MAPbI_3_ | Spiro-OMeTAD | None | Ag | / | / | 100 | 60 | 1.00 | / | 0.0919 | 1.07 | 20.10 | 67.00 | 14.60 | 10.1039/c8me00031j |
| Co-evaporation | ITO | C_60_ | None | MAPbI_3−x_Cl_x_ | TPTPA | MoOx | Al | / | / | 85 | 120 | 0.67 | 250.0 | 0.2 | 0.93 | 17.60 | 74.00 | 12.10 | 10.7567/JJAP.57.102303 |
| Co-evaporation | ITO | TiO_2_ | C_60_ | MAPbI₃ | TaTm | TaTm: F6-TCNNQ | Au | / | / | None | None | 1.00 | 95.0 | 0.04 | 1.19 | 12.53 | 81.54 | 12.16 | 10.1039/c8ee01936c |
| Co-evaporation | ITO | C60: PhIm | C_60_ | MAPbI₃ | TaTm | TaTm: F6-TCNNQ | Au | / | / | None | None | 1.00 | 420.0 | 0.04 | 1.13 | 18.66 | 83.31 | 17.55 | 10.1039/c8ee01936c |
| Co-evaporation | FTO | TiO_2_ | None | CsPbI_2_Br | Spiro-OMeTAD | None | Au | 0.88 | 1.24 | 300 | 1 | / | 400.0 | 0.09 | 1.10 | 10.90 | 49.00 | 5.70 | 10.1007/s13391-018-0095-1 |
| Co-evaporation | ITO | C_60_ | None | MAPbI₃ | PMA | None | Ag | / | / | 150 | 10 | / | 500.0 | / | 0.84 | 13.90 | 55.40 | 6.50 | 10.1039/c8dt03680b |
| Co-evaporation | ITO | C_60_ | None | MAPbI₃ | PMA | TaTm | Ag | / | / | None | None | / | / | / | 1.05 | 20.90 | 60.60 | 13.30 | 10.1039/c8dt03680b |
| Co-evaporation | ITO | PCBM | ICBA | MAPbBr_3_ | NiO | None | Ag | 0.2 | 0.2 | None | None | 0.10 | 93.0 | 0.042 | 1.54 | 5.38 | 76.00 | 6.03 | 10.1039/c8ee02575d |
| Co-evaporation | ITO | PCBM | ICBA | MAPbIBr_2_ | NiO | None | Ag | 0.1 | 0.2 | None | None | 0.10 | 122.0 | 0.042 | 1.45 | 5.43 | 77.00 | 6.07 | 10.1039/c8ee02575d |
| Co-evaporation | ITO | PCBM | ICBA | MAPbI_2_Br | NiO | None | Ag | 0.1 | 0.2 | None | None | 0.10 | 160.0 | 0.042 | 1.25 | 5.53 | 78.00 | 5.43 | 10.1039/c8ee02575d |
| Co-evaporation | ITO | ICBA | PCBM | MAPbI_3_ | NiO | None | Ag | 0.2 | 0.2 | None | None | 0.10 | 340.0 | 0.042 | 1.15 | 5.42 | 77.00 | 4.81 | 10.1039/c8ee02575d |
| Co-evaporation | ITO | C_60_ | None | MAPbI_3_ | m-MTDATA | None | Ag | / | / | None | None | 1.00 | 600.0 | / | 1.01 | 18.90 | 75.00 | 14.40 | 10.1039/c8tc05372c |
| Co-evaporation | ITO | C_60_ | None | MAPbI_3_ | TaTm | None | Ag | / | / | None | None | 1.00 | 600.0 | / | 1.06 | 20.10 | 76.00 | 16.30 | 10.1039/c8tc05372c |
| Co-evaporation | ITO | C_60_ | None | MAPbI_3_ | TcTa | None | Ag | / | / | None | None | 1.00 | 600.0 | / | 1.00 | 18.90 | 64.00 | 12.20 | 10.1039/c8tc05372c |
| Co-evaporation | ITO | C_60_ | None | MAPbI_3_ | NiOx | None | Au | / | 0.3 | 100 | 60 | 15.00 | / | 0.105 | 1.06 | 20.70 | 69.00 | 15.40 | 10.1002/aenm.201802995 |
| Co-evaporation | FTO | PCBM | None | MAPbI₃ | PTAA | None | Al | / | 0.75 | 100 | 60 | 76.66 | 350.0 | / | 1.03 | 22.00 | 77.00 | 17.40 | 10.1007/s10854-019-00842-y |
| Co-evaporation | FTO | C_60_ | None | MAPbI_3_ | Spiro-OMeTAD | None | Ag | / | 1.1 | None | None | / | 400.0 | 0.1 | 1.03 | 18.75 | 75.25 | 14.63 | 10.1088/1361-6463/ab070d |
| Co-evaporation | FTO | C_60_ | None | FAPbI_1.5_Br_1.5_ | spiro-OMeTAD | None | Ag | 1.2 | 0.4 | 150 | 20 | 1.00 | 250.0 | 0.09 | 1.08 | 19.90 | 68.00 | 14.61 | 10.1016/j.solmat.2019.02.014 |
| Co-evaporation | ITO | C_60_ | None | MAPbI_3_ | TPA-2,7-FLTPA-TPA | None | Ag | / | 0.1 | None | None | 1.00 | / | 0.04 | 1.05 | 20.82 | 78.00 | 17.10 | 10.1039/c9ta01681c |
| Co-evaporation | ITO | C_60_ | None | MAPbI_3_ | TPA-3,6-FLTPA-TPA | None | Ag | / | 0.1 | None | None | 1.00 | / | / | 1.05 | 19.85 | 67.00 | 13.90 | 10.1039/c9ta01681c |
| Co-evaporation | ITO | C_60_ | None | MAPbI_3_ | TaTm | None | Ag | / | 0.1 | None | None | 1.00 | / | / | 1.04 | 20.42 | 75.00 | 15.90 | 10.1039/c9ta01681c |
| Co-evaporation | ITO | C_60_ | None | CsPbI_3_ | PTAA | None | Cu | / | 0.0748 | None | None | / | 500.0 | 0.16 | 0.96 | 17.80 | 73.00 | 12.50 | 10.1002/aenm.201900555 |
| Co-evaporation | ITO | C_60_: PhIm | None | MAPbI_3_ | F6-TCNNQ | TaTm | Au | 1 | 0.5 | None | None | / | / | / | 0.88 | 18.30 | 80.00 | 12.80 | 10.1039/c9ta04367e |
| Co-evaporation | ITO | C_60_ | None | MAPbI_3_ | MoO3 | TaTm | Ag |  |  | None | None | 1.00 | 590.0 | 0.0651 | 1.08 | 21.70 | 82.50 | 19.30 | 10.1021/acs.chemmater.9b01396 |
| Co-evaporation | ITO | C_60_ | None | MAPbI_3_ | NiOx | None | Au |  | 0.3 | None | None | 18.00 | 250.0 | 0.105 | 1.03 | 20.70 | 79.00 | 16.80 | 10.1109/JPHOTOV.2019.2920727 |
| Co-evaporation | FTO | C_60_ | None | MAPbI_3_ | Spiro-OMeTAD | None | Ag | / | / | None | None | 50.00 | 810-850 | / | / | / | / | 15.70 | 10.1021/acsami.9b07619 |
| Co-evaporation | ITO | C_60_ | None | CsPbI₂Br | TAPC | TAPC: MoO₃ | Ag | 0.59 | 0.92 | 260 | 1 | 0.13 | 400.0 | 0.051 | 1.13 | 15.60 | 74.00 | 13.00 | 10.1002/adfm.201905163 |
| Co-evaporation | ITO | TiO₂ | None | MAPbI_3_ | TaTm: F6-TCNNQ | None | Au | 1 | 0.5 | None | None | 1.00 | 590.0 | 0.0653 | 1.16 | 20.80 | 81.20 | 19.70 | 10.1002/adma.201902692 |
| Co-evaporation | ITO | None | None | MAPbI₃ | MoO3 | None | Ag | 1 | 0.6 | 100° | 10 | 1.00 | 560.0 | 0.065 | 1.13 | 20.30 | 75.50 | 17.30 | 10.1002/ente.201900734 |
| Co-evaporation | ITO | C_60_ | None | MAPbI_3–x_Cl_x_ | TaTm | None | Ag | / | 0.6 | 100 | 5 | 1.00 | 500.0 | / | 1.12 | 19.00 | 73.00 | 16.10 | 10.1002/ente.201900784 |
| Co-evaporation | ITO | C_60_ | None | MAPbI₃ | MeO-2PACz | None | Cu | 3.5 | 1.4 | 100 | 45 | 1.00 | / | 0.16 | 1.15 | 22.60 | 75.40 | 19.60 | 10.1039/c9ee02268f |
| Co-evaporation | FTO | C_60_ | None | MAPbI_3_ | CuPc | F4-TCNQ | Au | / | 1.1 | 100 | 15 | 45.00 | 400.0 | 0.1 | 0.95 | 19.59 | 70.71 | 13.03 | 10.1088/1361-6528/ab4f2a |
| Co-evaporation | FTO | C_60_:(RuCp*mes)_2_ | None | MAPbI_3_ | CuPc | F4-TCNQ | Au | / | 1.1 | None | None | 1.00 | 500.0 | 0.0653 | 1.06 | 20.60 | 81.00 | 17.70 | 10.1039/c9ta09838k |
| Co-evaporation | FTO | None | None | MAPbI_3_ | CuPc | F4-TCNQ | Au | / | 1.1 | None | None | 1.00 | 500.0 | 0.0653 | 0.82 | 13.60 | 23.00 | 2.60 | 10.1039/c9ta09838k |
| Co-evaporation | ITO | PCBM | None | MAPbI₃ | PTTh | None | Au | / | / | 100 | 30 | 1.00 | 300.0 | 0.25 | 0.96 | 13.70 | 45.00 | 5.90 | 10.1021/acsami.9b20981 |
| Co-evaporation | ITO | None | None | MAPbI₃ | TaTm | None | Au | / | / | 100 | 30 | 1.00 | 300.0 | 0.25 | 0.97 | 18.20 | 46.00 | 8.10 | 10.1021/acsami.9b20981 |
| Co-evaporation | ITO | C_60_ | None | MAPbI₃ | TaTm | None | Ag | 1 | 0.6 | None | None | 1.00 | 600.0 | 0.05 | 1.12 | 19.50 | 80.00 | 18.00 | 10.3389/fchem.2019.00936 |
| Co-evaporation | FTO | C_60_ | None | MAPbI_3_ | Spiro-OMeTAD | None | Au | 0.4 | 0.4 | None | None | 1.00 | / | 0.0919 | / | / | / | 19.20 | 10.1002/aenm.201903653 |
| Co-evaporation | ITO | C_60_ | None | MAPbI_3_ | TaTm | None | Ag | 1 | 0.6 | None | None | 1.00 | 650.0 | 0.0651 | 1.13 | 20.50 | 77.80 | 18.10 | 10.1039/D0RA00214C |
| Co-evaporation | FTO | c-TiO₂ | None | CsPbBr₃ | None | None | C | 0.5 | 0.8 | 300 | 20 | 10.00 | 300.0 | 0.09 | 1.36 | 7.16 | 72.97 | 7.09 | 10.1039/d0ra00288g |
| Co-evaporation | ITO | C_60_ | None | Cs_0.1_FA_x_PbI_2+x_Br_0.1_ | Spiro-TTB: F6-TCNNQ | Spiro-TTB | Ag | 1.55 | 0.83 | 100 | 10 | 1.00 | 400.0 | 0.0644 | 1.07 | 19.50 | 79.70 | 16.60 | 10.1039/D0TC01550D |
| Sequential evaporation | ITO | C_60_ | None | MAPbI_3_ | Spiro-TTB: F6-TCNNQ | Spiro-TTB | Ag | / | / | 100 | 30 | 6650.00 | 350.0 | 0.09 | 0.80 | 13.60 | 50.00 | 5.40 | 10.1039/C4RA03820G |
| Sequential evaporation | FTO | TiO₂ | None | Cs_0.15_FA_0.85_PbI_3_ | NPB | MoO3 | Au | / | / | 60 | 30 | 3.50 | 560.0 | 0.09 | 1.07 | 23.35 | 75.40 | 18.89 | 10.1039/D1EE00634G |
| Sequential evaporation | ITO | ZnO | None | CsPbBr_3_ | Spiro-OMeTAD | None | Au | / | / | 150 | 5 | / | 400.0 | 0.01 | 1.30 | 5.46 | 70.44 | 5.00 | 10.1021/acsami.1c01674 |
| Sequential evaporation | FTO | TiO_2_ | None | CsPbBr_3_ | P3HT | None | Au | / | / | 450 | 20 | / | 650.0 | 0.106 | 1.64 | 6.72 | 71.00 | 7.90 | 10.1016/j.jallcom.2022.163725 |
| Sequential evaporation | FTO | SnO_2_ | None | CsPbBr_3_ | None | None | C | 4 | 4 | 300 | 5 | / | 560.0 | 0.1475 | 1.55 | 7.95 | 82.00 | 10.09 | 10.1021/acsaem.2c00506 |
| Sequential evaporation | FTO | SnO_2_ | None | Cs_0.05_FA_0.95_PbI_3_ | Spiro-OMeTAD | None | Au | / | / | 170 | 15 | / | / | 0.1 | 1.15 | 25.92 | 81.78 | 24.42 | 10.1126/sciadv.abo7422 |
| Sequential evaporation | FTO | TiO_2_ | None | CsPbI_2_Br | CuSCN | None | Au | 0.8 | 3 | 300 | 20 | 1.00 | 420.0 | / | 1.05 | 12.30 | 76.00 | 9.96 | 10.1021/acsaem.2c01155 |
| Sequential evaporation | ITO | SnO_2_ | None | FAPbI_3_ | spiro-OMeTAD | None | Au | / | 1.5 | 150 | 15 | 5.00 | / | 0.1 | 1.07 | 24.90 | 75.40 | 20.19 | 10.1002/adfm.202208392 |
| Sequential evaporation | ITO | SnO_2_ | None | FAPbI_3_ | spiro-OMeTAD | None | Au | / | 1.5 | 150 | 15 | 5.00 | / | 1 | 1.06 | 24.13 | 73.60 | 18.91 | 10.1002/adfm.202208392 |
| Sequential evaporation | FTO | SnO_2_ | None | FA_0.9_Cs_0.1_PbI_2.454_Br_0.399_Cl_0.147_ | Spiro-OMeTAD | None | Au | / | / | 170 | 30 | 6.00 | 490.0 | 0.1 | 1.16 | 25.52 | 82.20 | 24.36 | 10.1002/adfm.202211232 |
| Sequential evaporation | FTO | SnO_2_ | None | FA_0.9_Cs_0.1_PbI_2.454_Br_0.399_Cl_0.147_ | spiro-OMeTAD | None | Au | / | / | 170 | 30 | 6.00 | 490.0 | 1 | 1.11 | 25.92 | 81.04 | 23.30 | 10.1002/adfm.202211232 |
| Sequential evaporation | ITO | SnO_2_ | None | FAPbI_3_ | spiro-OMeTAD | None | Au | / | / | 160 | 20 | 1.00 | 400.0 | 0.1 | 1.14 | 24.36 | 77.30 | 21.40 | 10.1002/smll.202307960 |
| Sequential evaporation | ITO | SnO_2_ | None | CsFAPbI_3_ | spiro-OMeTAD | None | Au | / | / | 160 | 20 | 1.00 | 400.0 | 0.1 | 1.09 | 25.31 | 79.70 | 22.00 | 10.1002/smll.202307960 |
| Sequential evaporation | FTO | SnO_2_ | None | Cs_0.05_FA_0.95_PbI_3_ | T2 | None | Au | 1 | 3 | 150 | 15 | / | 350.0 | 0.1 | 1.18 | 26.47 | 84.94 | 26.21 | 10.1016/j.joule.2024.02.019 |
| Sequential evaporation | ITO | C_60_ | None | CsPbI_2_Br | PTAA | None | Ag | 2 | 5 | 300 | 2 | 4.00 | 400.0 | 0.135 | 1.20 | 12.78 | 69.32 | 13.41 | 10.1021/acsnano.4c03079 |
| Evaporation-gas | FTO | C_60_ | None | Cs_0.14_FA_0.86_Pb(Br_x_I_1-x_)_3_ | NiOx | None | Ag | 0.4 | 4 | None | None | / | / | 0.147 | 1.04 | 21.97 | 77.00 | 17.96 | 10.1039/D2RA01360F |
| Evaporation-gas | ITO | SnO_2_ | None | MAPbInI_3_ | spiro-OMeTAD | None | Au | / | 0.5 | None | None | 0.10 | 500.0 | 0.16 | 1.17 | 23.20 | 78.00 | 21.20 | 10.1002/solr.202200106 |
| Evaporation-gas | FTO | TiO_2_ | None | Cs_x_FA_1−x_PbI_3−y_Br_y_ | Spiro-OMeTAD | None | Au | 0.3 | 4 | 170 | 10 | 0.08 | / | 3 | 1.00 | 21.94 | 78.24 | 17.10 | 10.1021/acsomega.3c00318 |
| Evaporation-gas | FTO | SnO_x_ | None | Cs_x_FA_1–x_PbI_3_ | Spiro-OMeTAD | None | Au | 0.4 | 4 | 150 | 10 | 8.00 | 577.5 | 0.147 | 1.11 | 23.64 | 79.00 | 20.79 | 10.1002/solr.202300062 |
| Evaporation-gas | FTO | SnO_x_ | None | Cs_0.24_FA_0.76_PbI_3_ | Spiro-OMeTAD | None | Au | 0.4 | 4 | 160 | / | / | / | 0.147 | 1.10 | 24.17 | 80.00 | 21.27 | 10.1021/acsaem.3c00676 |
| Evaporation-gas | FTO | SnO_2_ | None | Cs_x_FA_1-x_PbI_3-y_Br_y_ | spiro-OMeTAD | None | Au | 0.4 | 4 | None | None | / | 577.5 | 0.16 | 1.14 | 23.20 | 83.00 | 21.90 | 10.1002/adma.202304625 |
| Evaporation-gas | FTO | SnO_x_ | None | Cs_x_FA_(1-x)_PbI_3_ | Spiro-OMeTAD | None | Au | 0.4 | 4 | 170 | 15 | 8.00 | / | 0.1475 | 1.14 | 23.82 | 81.00 | 22.06 | 10.1039/d3ta04666d |
| Evaporation-gas | FTO | SnO_2_ | None | FAPbI_3_ | Spiro-OMeTAD | None | Au | / | 3.5 | None | None | 8.00 | 350.0 | 0.148 | 1.16 | 24.00 | 79.20 | 22.11 | 10.1002/adfm.202313435 |
| Evaporation-gas | FTO | SnO_2_ | None | FAPbI_3_ | Spiro-OMeTAD | None | Au | / | 3.5 | None | None | 8.00 | 350.0 | 1 | 1.12 | 23.27 | 78.70 | 20.60 | 10.1002/adfm.202313435 |
| Evaporation-gas | ITO | SnO_x_ | C_60_ | FA_0.9_Cs_0.1_PbI_3-X_Cl_X_ | TaTm | TaTm: F6TCNNQ | Au | / | / | None | None | / | 210.0 | 0.05 | 1.03 | 24.06 | 75.30 | 18.70 | 10.1021/acsenergylett.3c02794 |
| Evaporation-gas | FTO | SnO_2_ | None | Cs_0.2_FA_0.8_PbI_3-x_Br_x_ | Spiro-OMeTAD | None | Au | / | / | None | None | 0.80 | 495.0 | 0.16 | 1.18 | 24.39 | 79.00 | 22.70 | 10.1002/adma.202401416 |
| Evaporation-gas | FTO | SnO_2_ | None | Cs_x_FA_1−x_PbI_3−y_Br_y_ | spiro-OMeTAD | None | Au | 0.4 | 4 | None | None | / | 600.0 | 0.1475 | 1.09 | 23.26 | 83.00 | 21.09 | 10.1039/d4se00320a |
| Evaporation-gas | FTO | SnO_x_ | None | Cs_0.14_FA_0.86_PbI_3-x_Br_x_ | Spiro-OMeTAD | None | Au | 0.4 | 4 | 170 | 15 | 0.80 | 557.5 | 0.1475 | 1.15 | 23.46 | 79.00 | 21.29 | 10.1021/acsaem.4c00080 |
| Evaporation-gas | FTO | C_60_ | None | Cs_x_FA_1-x_PbI_y_Br_3-y_ | MeO-2PACz | None | Ag | 0.4 | 4 | None | None | 8.00 | / | 0.1475 | 1.13 | 23.76 | 80.00 | 21.53 | 10.1002/smtd.202401339 |
| Evaporation-gas | FTO | SnO_2_ | None | Cs_0.16_FA_0.84_PbI_2.84_Br_0.16_ | spiro-OMeTAD | None | Au | 0.4 | 4 | 160 | 5 | / | 600.0 | 1 | 1.17 | 23.20 | 81.00 | 22.10 | 10.1002/adma.202412021 |
| Evaporation-gas | FTO | SnO_2_ | None | Cs_0.12_FA_0.88_PbI_3_ | Spiro-OMeTAD | None | Au | 0.4 | 4 | 170 | 60 | / | 300.0 | 0.148 | 1.12 | 24.58 | 78.00 | 21.54 | 10.1016/j.cej.2024.156259 |
| Single-source evaporation | / | PCBM | None | MAPbI_3_ | PEDOT: PSS | None | Ag | / | / | 140 | 20 | / | 500.0 | 100 | 0.89 | 12.80 | 67.79 | 7.73 | 10.3390/coatings8080256 |
| Single-source evaporation | FTO | PCBM | None | FAPbI_3_ | spiro-OMeTAD | None | Au | / | / | None | None | 50.00 | / | 36 | 1.08 | 17.19 | 67.60 | 12.55 | 10.1007/s10854-019-01155-w |
| Single-source evaporation | ITO | PCBM | None | MAPbI_3_ | PEDOT: PSS | None | Ag | / | / | 150 | 20 | 15.00 | / | 0.1 | 0.94 | 19.97 | 53.00 | 9.92 | 10.3390/ma12081237 |
| Single-source evaporation | FTO | c-TiO_2_ | None | CsPbBr_3_ | Spiro-OMeTAD | None | Au | / | / | None | None | / | 650.0 | 0.09 | 1.37 | 7.79 | 81.00 | 8.65 | 10.1016/j.jallcom.2019.152903 |
| Single-source evaporation | ITO | SnO_2_ | None | MA_0.55_FA_0.45_PbI_3_ | Spiro-OMeTAD | None | Au | / | / | None | None | / | 450.0 | 0.01 | 1.00 | 20.00 | 70.00 | 14.00 | 10.1002/adfm.202300588 |
| Single-source evaporation | ITO | SnO_2_ | None | CsPb(I_0.83_Br_0.17_)_3_ | Spiro-OMeTAD | None | Au | / | / | 330 | 1 | 15.00 | 350.0 | 0.06 | 1.17 | 16.80 | 76.00 | 14.90 | 10.1039/d3ta05881f |
| Single-source evaporation | ITO | C_60_ | None | MA_1-x_FA_x_PbI_3_(Cl)_y_ | 2PACz | None | Ag | 1 | / | 100 | 10 | / | 400.0 | 0.0633 | 1.16 | 21.60 | 79.40 | 19.70 | 10.1016/j.joule.2024.09.001 |
| Evaporation-solution | FTO | c-SnO_2_ | None | MAPbI_x_Br_3-x_ | Spiro-OMeTAD | None | Au | / | 5 | 110 | 60 | / | 350.0 | 0.07 | 1.07 | 22.50 | 75.60 | 18.20 | 10.1186/s11671-020-03359-0 |
| Evaporation-solution | ITO | C_60_ | None | MAPb(I_1-x-y_Br_x_ Cl_y_) | TaTm | MoOx | Ag | / | 2.5 | 100 | 45 | 6.65 | 350.0 | 0.06 | 1.08 | 22.10 | 79.60 | 19.10 | 10.1021/acsaem.0c00686 |
| Evaporation-solution | ITO | PCBM | None | MAPbI_3_ | PEDOT: PSS | None | Ag | / | 0.1 | 100 | 20 | / | 110.0 | / | 0.85 | 14.30 | 77.73 | 9.44 | 10.1007/s13391-020-00250-z |
| Evaporation-solution | IOH | SnO_2_ | None | Cs_0.05_(FA_0.83_MA_0.17_)_0.95_Pb(I_0.83_Br_0.17_)_3_ | Spiro-OMeTAD | None | Au | 1 | 7 | 150 | 15 | 6.00 | 560.0 | 0.1 | 1.16 | 23.31 | 72.00 | 18.70 | 10.1039/d1ta07579a |
| Evaporation-solution | FTO | SnO_2_ | None | FA_0.995_MA_0.005_Pb(I_0.995_Br_0.005_)_3_ | spiro-OMeTAD | None | Au | / | 0.5 | 150 | 15 | 1.00 | 600.0 | 0.0784 | 1.20 | 24.90 | 84.30 | 24.30 | 10.1039/d1ee02897a |
| Evaporation-solution | FTO | p-SnO_2_ | PCBM | MAPbI_3_ | Spiro-OMeTAD | None | Au | / | / | 100 | 30 | 10.60 | 750.0 | 0.086 | 1.05 | 20.00 | 74.00 | 16.00 | 10.1039/D1SE01692J |
| Evaporation-solution | FTO | p-SnO_2_ | PCBM | MAPb(Br_0.18_I_0.82_)_3_ | Spiro-OMeTAD | None | Au | / | / | 100 | 30 | 10.60 | 750.0 | 0.086 | 1.08 | 17.00 | 74.00 | 14.00 | 10.1039/D1SE01692J |
| Evaporation-solution | FTO | SnO_2_ | None | FAPbI_3_ | Spiro-OMeTAD | None | Au | 0.1 | 4 | 150 | 30 | 50.00 | 380.0 | 0.1 | 1.17 | 24.57 | 79.85 | 22.88 | 10.1002/adma.202205027 |
| Evaporation-solution | FTO | SnO_2_ | None | (FA)_0.975_(Cs)_0.025_PbI_3_ | Spiro-OMeTAD | None | Au | 0.1 | 4 | 150 | 30 | 50.00 | 380.0 | 0.1 | 1.16 | 24.58 | 80.83 | 23.09 | 10.1002/adma.202205027 |
| Evaporation-solution | FTO | SnO_2_ | None | (FA)_0.95_(Cs)_0.05_PbI_3_ | Spiro-OMeTAD | None | Au | 0.1 | 4 | 150 | 30 | 50.00 | 380.0 | 0.1 | 1.20 | 24.23 | 83.11 | 24.17 | 10.1002/adma.202205027 |
| Evaporation-solution | FTO | SnO_2_ | None | (FA)_0.9_(Cs)_0.1_PbI_3_ | Spiro-OMeTAD | None | Au | 0.1 | 4 | 150 | 30 | 50.00 | 380.0 | 0.1 | 1.17 | 24.39 | 82.21 | 23.51 | 10.1002/adma.202205027 |
| Evaporation-solution | ITO | SnO_2_ | None | (FAPbI_3_)_0.91_(MAPbBr_3_)_0.09_ | Spiro-OMeTAD | None | Au | / | 1 | 150 | 15 | 5.00 | 200.0 | 0.1 | 1.10 | 24.06 | 74.59 | 19.80 | 10.1016/j.jallcom.2023.170255 |
| Evaporation-solution | ITO | C_60_ | None | Cs_0.05_MA_0.28_FA_0.67_Pb(Br_0.03_Cl_0.06_I_0.91_)_3_ | 2PACz | None | Ag | / | 1 | 150 | 15 | / | / | 0.105 | 1.10 | 22.49 | 80.00 | 18.20 | 10.1002/solr.202400165 |
| Evaporation-solution | FTO | C_60_ | None | CsₓFAₓMAₓPbI₃ | NiOx | None | Cu | / | / | 150 | 20 | / | / | 61.56 | 12.05 | 2.11 | 80.87 | 20.56 | 10.1007/s40820‑024‑01408‑2 |
| Evaporation-solution | ITO | C_60_ | None | FAMAPbI–BrCl | MeO-2PACz | None | Ag | / | 5 | 150 | 15 | 5.00 | / | 0.1 | 1.18 | 25.33 | 82.03 | 24.43 | 10.1039/d4ee02718c |
| Evaporation-solution | ITO | C_60_ | None | FAPbI-BrCl | MeO-2PACz | None | Ag | / | 5 | 150 | 15 | 5.00 | / | 0.1 | 1.10 | 24.26 | 76.60 | 20.37 | 10.1039/d4ee02718c |
| Evaporation-solution | ITO | C_60_ | None | FAMAPbI | MeO-2PACz | None | Ag | / | 5 | 150 | 15 | 5.00 | / | 0.1 | 0.78 | 16.90 | 54.64 | 8.82 | 10.1039/d4ee02718c |
| Evaporation-solution | ITO | C_60_ | None | FAMAPbI-Cl | MeO-2PACz | None | Ag | / | 5 | 150 | 15 | 5.00 | / | 0.1 | 1.10 | 23.77 | 75.00 | 19.67 | 10.1039/d4ee02718c |
| Evaporation-solution | ITO | C_60_ | None | MAPbI_3_ | spiro-OMeTAD | None | Au | / | 20 | 150 | 10 | 5.00 | / | 0.16 | 1.01 | 18.10 | 70.00 | 12.60 | 10.1038/s41598-017-18970-y |
| Evaporation-solution | ITO | C_60_ | None | MAPbI_3_ | spiro-OMeTAD | None | Au | / | 20 | 150 | 10 | 5.00 | / | 1.2 | 1.01 | 17.60 | 60.00 | 11.60 | 10.1038/s41598-017-18970-y |
| Evaporation-solution | ITO | C_60_ | None | MAPbI_3_ | spiro-OMeTAD | None | Au | / | 20 | 150 | 10 | 5.00 | / | 16 | 4.90 | 3.30 | 50.00 | 7.80 | 10.1038/s41598-017-18970-y |
| Evaporation-solution | ITO | PCBM | None | MAPbI_3_ | PTAA | None | AZO/MgF2 | / | 1.25 | 100 | 10 | 0.05 | 520.0 | 0.273 | 1.12 | 19.90 | 75.70 | 16.80 | 10.1002/advs.201700675 |
| Evaporation-solution | ITO | C_60_ | None | MAPbI_3_ | Vox | CuPc | Ag | / | 2 | 60 | 5 | 10.00 | 200.0 | 0.0706 | 1.00 | 21.68 | 73.80 | 15.98 | 10.1039/c8cc03672a |
| Evaporation-solution | ITO | C_60_ | None | CsxFA_1−x_Pb(I,Br)_3_ | spiro-TTB | None | Ag | 0.1 | 1 | 150 | 30 | 5.00 | 440.0 | 1.42 | 1.79 | 19.53 | 73.10 | 25.52 | 10.1038/s41563-018-0115-4 |
| Evaporation-solution | ITO | C_60_ | None | MAPbI_3_ | Spiro-OMeTAD | None | Au | / | 20 | 150 | 10 | / | / | 0.16 | 0.98 | 19.10 | 74.00 | 13.87 | 10.1021/acsaem.8b00803 |
| Evaporation-solution | FTO | TiO2 | None | MAPbI_3_ | Spiro-OMeTAD | None | Au | / | 2 | 120 | 25 | 3.99 | / | 0.1 | 1.09 | 20.30 | 72.00 | 15.93 | 10.1021/acs.jpcc.8b08075 |
| Evaporation-solution | FTO | TiO_2_ | None | MAPbI_3_ | Spiro-OMeTAD | None | Au | / | 2 | 120 | 25 | 3.99 | / | 0.1 | 0.98 | 19.70 | 55.00 | 10.62 | 10.1021/acs.jpcc.8b08075 |
| Evaporation-solution | FTO | TiO_2_ | None | MAPbI_3_ | CuI | None | Au | / | / | None | None | / | / | / | 0.71 | 16.47 | 39.00 | 4.56 | 10.1016/j.scib.2018.11.004 |
| Evaporation-solution | flexible foil | C_60_ | None | MAPbI_3_ | Spiro-OMeTAD | None | Au | / | 1.4 | 100 | 30 | 0.04 | 120.0 | 0.15 | 1.07 | 19.70 | 74.00 | 15.80 | 10.1038/s41427-018-0099-1 |
| Evaporation-solution | FTO | TiO_2_ | None | MAPbI_3_ | Spiro-OMeTAD | None | Au | / | 0.5 | 100 | 90 | 1.00 | / | 0.16 | 1.09 | 22.70 | 69.90 | 17.30 | 10.1021/acsami.8b16963 |
| Evaporation-solution | FTO | TiO_2_ | None | MAPbI_3-x_Cl_x_ | Spiro-OMeTAD | None | Au | / | / | 100 | 120 | / | 400.0 | / | 1.05 | 23.62 | 76.80 | 19.10 | 10.1021/acsami.9b05101 |
| Evaporation-solution | ITO | PCBM | None | (FA,MA)Pb(I,Br)₃ | PTAA | None | ZnO | / | 1.25 | None | None | 0.05 | / | 0.25 | 1.02 | 19.30 | 69.80 | 13.80 | 10.1039/c9ee02043h |
| Solution-evaporation | ITO | SnO_2_ | None | Cs_0.05_FA_0.5_MA_0.45_PbI_3_ | Spiro-OMeTAD | None | Au | / | / | None | None | 1.00 | 680.0 | 0.09 | 1.12 | 22.73 | 74.00 | 18.81 | 10.1002/aenm.202300153 |
| Solution-evaporation | ITO | SnO_2_ | None | CsxFA1-xPbX_3_ | spiro-OMeTAD | MoO3 | Ag | 4.5 | / | None | None | 100.00 | 350.0 | 0.1 | 1.16 | 25.30 | 82.11 | 24.10 | 10.1016/j.joule.2022.05.002 |
| Solution-evaporation | FTO | c-TiO_2_ | None | CsPbIBr_2_ | CuPc | None | C | 2 | / | 280 | 10 | 9.00 | / | 0.071 | 1.29 | 10.40 | 65.30 | 8.76 | 10.1016/j.electacta.2019.135266 |
| Solution-evaporation | FTO | c-TiO_2_ | m-TiO_2_ | CsPbBr_3_ | MnS | None | C | / | / | None | None | / | / | / | 1.52 | 8.28 | 83.00 | 10.45 | 10.1021/acsami.9b06356 |
| Solution-evaporation | ITO | SnO_2_ | None | CsPbI₂Br | Spiro-OMeTAD | None | Au | / | / | None | None | / | / | 0.09 | 1.27 | 16.72 | 77.18 | 16.37 | 10.1002/solr.201900254 |
| Solution-evaporation | FTO | c-TiO_2_ | m-TiO_2_ | MAPb(I_1-x_Brₓ)₃ | spiro-OMeTAD | None | Au | / | / | 100 | 10 | / | / | 0.07 | 1.04 | 21.97 | 67.60 | 15.40 | 10.1021/acsami.9b06673 |
| Solution-evaporation | FTO | c-TiO_2_ | None | MAPbI_3_ | spiro-OMeTAD | None | Au | 0.31 | 0.24 | None | None | 6.40 | 150.0 | 0.1 | 1.02 | 21.31 | 68.90 | 16.40 | 10.1002/advs.201802046 |
| Solution-evaporation | FTO | TiO_2_ | None | MAPb(I, Cl)_3_ | spiro-OMeTAD | None | Ag | / | / | None | None | 800.00 | / | 0.1 | 1.03 | 20.50 | 73.34 | 15.57 | 10.1038/s41598-017-14920-w |
| Solution-evaporation | FTO | c-TiO_2_ | m-TiO_2_ | MAPbI₃ | spiro-MeOTAD | None | Au | / | / | 100 | 20 | 100.00 | / | 0.1 | / | / | / | 17.20 | 10.1021/acs.jpclett.7b01508 |
| Evaporation-gas | FTO | SnO₂ | None | Cs₀.₂₄FA₀.₇₆PbI_3-x_Br_x_ | spiro-OMeTAD | None | Au | 0.4 | 0.1 | None | None | / | 471.0 | / | 1.02 | 21.94 | 78.90 | 17.66 | 10.1039/d0se00088d |
| Evaporation-gas | FTO | TiO_2_ | None | MAPbI_3_ | Spiro-OMeTAD | None | Au | / | 1 | None | None | 5.00 | / | 0.045 | 1.01 | 23.66 | 72.40 | 17.30 | 10.1016/j.nanoen.2019.03.014 |

**Table S3.** The 23 input features and corresponding description of ML models

| Features | Description |
| --- | --- |
| Method | Co-evaporation, Sequential vapor deposition, Evaporation-gas, Single-source evaporation, Evaporation-solution and Solution-evaporation |
| Sub_WF | Work function of substrate materials |
| ETL_HOMO_1 | HOMO of the first ETL layer |
| ETL_LUMO_1 | LUMO of the first ETL layer |
| ETL_HOMO_2 | HOMO of the second ETL layer |
| ETL_LUMO_2 | LUMO of the second ETL layer |
| MA_ratio | Ratio of MA cations to all cations in the perovskites |
| FA_ratio | Ratio of FA cations to all cations in the perovskites |
| Cs_ratio | Ratio of Cs cations to all cations in the perovskites |
| I_ratio | Ratio of I anions to all anions in the perovskites |
| Br_ratio | Ratio of Br anions to all anions in the perovskites |
| HTL_HOMO_1 | HOMO of the first HTL layer |
| HTL_LUMO_1 | LUMO of the first HTL layer |
| HTL_HOMO_2 | HOMO of the second HTL layer |
| HTL_LUMO_2 | LUMO of the second HTL layer |
| Ele_WF | Work function of back electrode materials |
| AX_evaporation rate | The evaporation rate of halogenated cations in perovskite precursors |
| BX2_ evaporation rate | The evaporation rate of halogenated lead in perovskite precursors |
| Annealing temperature | Annealing temperature of perovskite films |
| Annealing time | Annealing time of perovskite films |
| Pressure | Chamber pressure for the evaporation of perovskite precursors |
| Perovskite thickness | The thickness of the formed perovskite film |
| Device area | The mask area of the *J*-*V* test of the device |

**Table S4.** Best parameters of 10 ML algorithms for PCE prediction.

| ML algorithms | Best parameters |
| --- | --- |
| LR | 'regressor__alpha': 1,  'regressor__solver': 'svd' |
| Linear SVR | 'regressor__C': 10.0, 'regressor__dual': True, 'regressor__epsilon': 0.1,  'regressor__loss': 'squared_epsilon_insensitive' |
| SVR | 'regressor__C': 10, 'regressor__epsilon': 0.2,  'regressor__gamma': 'scale', 'regressor__kernel': 'rbf' |
| DTree | 'regressor__max_depth': None,  'regressor__max_features': None, 'regressor__min_samples_leaf': 4, 'regressor__min_samples_split': 2 |
| ETree | 'regressor__max_depth': None,  'regressor__max_features': 'sqrt',  'regressor__min_samples_leaf': 1, 'regressor__min_samples_split': 2,  'regressor__n_estimators': 100 |
| RF | 'regressor__bootstrap': False,  'regressor__max_depth': None,  'regressor__max_features': 'sqrt',  'regressor__min_samples_leaf': 1, 'regressor__min_samples_split': 2,  'regressor__n_estimators': 200 |
| GBoost | 'regressor__learning_rate': 0.1, 'regressor__max_depth': 7, 'regressor__max_features': 'log2', 'regressor__min_samples_leaf': 2, 'regressor__min_samples_split': 2, 'regressor__n_estimators': 200 |
| MLP | 'regressor__activation': 'tanh',  'regressor__alpha': 0.0001,  'regressor__hidden_layer_sizes': (50,), 'regressor__learning_rate': 'constant',  'regressor__solver': 'adam' |
| AdaBoost | 'regressor__base_estimator__max_depth': 5,  'regressor__learning_rate': 1.0,  'regressor__n_estimators': 200 |
| XGBoost | 'regressor__colsample_bytree': 0.8, 'regressor__gamma': 0, 'regressor__learning_rate': 0.05, 'regressor__max_depth': 7, 'regressor__n_estimators': 200, 'regressor__subsample': 0.9 |

**Table S5.** The performance of 10 ML algorithms corrected by experimental data on the training and testing sets.

| ML algorithms | Train set | | | Test set | | |
| --- | --- | --- | --- | --- | --- | --- |
|  | *R*^2^ | RMSE(%) | MAPE(%) | *R*^2^ | RMSE(%) | MAPE(%) |
| LR | 0.6139 | 3.2930 | 25.0488 | 0.6314 | 3.3391 | 25.6799 |
| Linear SVR | 0.5748 | 3.4558 | 24.6254 | 0.6174 | 3.4021 | 24.1916 |
| SVR | 0.8695 | 1.9146 | 10.7291 | 0.8662 | 2.0119 | 12.1733 |
| DTree | 0.9793 | 0.7622 | 3.5747 | 0.8426 | 2.1820 | 12.1911 |
| ETree | 0.9993 | 0.1355 | 0.0563 | 0.9464 | 1.2730 | 8.3318 |
| RF | 0.9993 | 0.1355 | 0.0567 | 0.9254 | 1.5025 | 10.0836 |
| GBoost | 0.9989 | 0.1776 | 0.4733 | 0.9256 | 1.5000 | 10.0827 |
| MLP | 0.9587 | 1.0776 | 6.2105 | 0.9439 | 1.3023 | 8.2239 |
| AdaBoost | 0.8915 | 1.7460 | 13.6870 | 0.8191 | 2.3390 | 18.5560 |
| XGBoost | 0.9972 | 0.2811 | 1.4414 | 0.9165 | 1.5890 | 10.7199 |

**Table S6.** The additional datasets during the period of 2024-2025.

| Method | Substrate | ETL-1 | ETL-2 | Perovskite | HTL-1 | HTL-2 | Back contact | AX ratio(Å/s) | BX_2_ ratio (Å/s) | Annealing temperature (°C) | Annealing time (min) | pressure/mbar(10⁻⁶) | thickness (mn) | Area (cm²) | PCE（%） | reference |
| --- | --- | --- | --- | --- | --- | --- | --- | --- | --- | --- | --- | --- | --- | --- | --- | --- |
| Evaporation-solution | ITO | C₆₀ | None | CsxFA1-xPb(IₓBr₁₋ₓ)₃ | Me-4PACz | None | Ag | / | / | 120 | 8 | / | / | 0.05 | 16.64 | 10.1038/s41467-025-61404-x |
| Evaporation-gas | ITO | C₆₀ | None | RbFAPbI_3_ | MeO-2PACz | None | Ag | / | 0.3 | 25 | 0 | 20 | / | 0.1125 | 21.7 | 10.1016/j.joule.2024.02.007 |
| Evaporation-solution | ITO | C₆₀ | None | CsxFA1-xPb(IₓBr₁₋ₓ)₃ | Me-4PACz | MeO-2PACz | Cu | 0.1 | 2 | 150 | 20 | 5 | / | 0.07 | 23.21 | 10.1002/aenm.202404954 |
| Evaporation-solution | ITO | C₆₀ | None | Cs_0.05_MA_0.28_FA_0.67_Pb(Br_0.03_Cl_0.06_I_0.91_)_3_ | 2PACz | None | Ag | / | / | 70 | 1 | / | 500 | 0.105 | 18.2 | 10.1002/solr.202400165 |
| Evaporation-gas | ITO | SnO₂ | C₆₀ | Cs_x_FA_1-x_PbIBr(SCN) | MeO-2PACz | None | Ag | / | / | 150 | 9 | / | / | 6.25 | 16.46 | 10.1002/aenm.202303423 |
| Evaporation-gas | FTO | C₆₀ | None | Cs_x_FA_1-x_PbI_3-y_Br_y_ | MeO-2PACz | None | Ag | / | 4 | 170 | 5 | / | / | 0.1475 | 19.48 | 10.1002/smtd.202401339 |
| Solution-evaporation | FTO | SnO₂ | None | FA_1−x_Cs_x_PbI_3−y_Br_y_ | Spiro-OMeTAD | None | Au | / | / | None | None | / | / | 0.1475 | 21.19 | 10.1002/solr.202400016 |
| Solution-evaporation | FTO | SnO₂ | None | Cs_x_FA_1−x_PbI_3-y_Br_y_ | Spiro-OMeTAD | None | Au | / | / | None | None | / | / | 0.1475 | 21.13 | 10.1016/j.mtener.2024.101540 |
| Evaporation-gas | ITO | C₆₀ | SnO₂ | FA_x_Cs_1-x_Pb(I_y_Br_1-y_)_3_ | NiO_X_ | MeO-2PACz | Ag | / | 4 | 170 | 5 | 8 | / | 1 | 18.7 | 10.1002/aenm.202405377 |
| Evaporation-gas | FTO | C₆₀ | None | CsFAPbI_3-y_Br_y_ | MeO-2PACz | None | Ag | / | 4 | None | None | / | / | 0.1475 | 19.51 | 10.1002/solr.202500053 |
| Co-evaporation | ITO | C₆₀ | None | Cs_0.2_FA_0.8_Pb(I_0.8_Br_0.2_)_3_ | CS90112 | TaTm | Ag | 0.45 | 0.35 | None | None | 8 | / | 0.05 | 17.5 | 10.1039/d5el00021a |

**Table S7.** Comparison of prediction performance evaluation parameters of the model trained based on the original test set and the new test set containing additional data.

| ML algorithms | Original test set | | | Test set with additional data | | |
| --- | --- | --- | --- | --- | --- | --- |
|  | *R*^2^ | RMSE(%) | MAPE(%) | *R*^2^ | RMSE(%) | MAPE(%) |
| ETree | 0.9464 | 1.2730 | 8.3318 | 0.9400 | 1.3454 | 8.3441 |

**Table S8.** Dataset of the latest advance PSCs fabricated by six different vapor deposition methods.

| Method | Substrate | ETL-1 | ETL-2 | Perovskite | HTL-1 | HTL-2 | Back electrode | AX evaporation ratio (Å/s) | BX_2_ evaporation ratio (Å/s) | Annealing temperature (℃) | Annealing time (min) | pressure/mbar (10^-6) | thickness (nm) | Area (cm²) | PCE（%） | reference |
| --- | --- | --- | --- | --- | --- | --- | --- | --- | --- | --- | --- | --- | --- | --- | --- | --- |
| Single-source evaporation | ITO | C_60_ | None | Cs_0.2_FA_0.8_PbI_3.1_ | MeO-2PACz | None | Ag | 2.89 | 2.89 | 135 | 25 | / | 650 | 0.01 | 14.06 | Adv. Energy Mater. 2025, 2406033 |
| Co-evaporation | ITO | C_60_ | None | FA_0.94_Cs_0.2_Pb(I_0.94_Br_0.11_)_3_ | MeO-2PACz | None | Ag | / | / | 180 | 5 | / | 540 | 0.1 | 18.8 | ACS Energy Lett. 2024, 9, 5639−5646 |
| Sequential vapor deposition | ITO | PCBM | None | Cs_y_FA_1-y_Pb(I_1-x_Cl_x_)_3_ | MeO-2PACz | None | Ag | / | / | 150 | 20 | 38 | 620 | 0.1 | 23 | Adv. Mater. 2025, 2501162 |
| Evaporation-gas | FTO | C_60_ | None | Cs_0.24_FA_0.76_Pb(I_0.8_Br_0.2_)_3_ | MeO-2PACz | None | Ag | 0.4 | 4.5 | 100 | 5 | 8 | / | 0.1475 | 19.35 | Nano Energy 139 (2025) 110914 |
| Evaporation-solution | ITO | PCBM | C_60_ | Cs_0.15_FA_0.85_PbI_1.8_Br_1.2_ | Me-4PACZ | None | Ag | 0.6 | 4 | 130 | 15 | 4 | 500 | 0.05 | 15.03 | ACS Nano 2025, 19, 15820−15830 |
| Solution-evaporation | FTO | TiO_2_ | None | MA_x_FA_1-x_PbI_3-y_Br_y_ | Spiro-OMeTAD | None | Ag | / | / | None | None | / | / | 0.12 | 17.94 | ACS Appl. Energy Mater. 2024, 7, 11225−11232 |

**Table S9.** The optimal device structure, process parameters and the highest predicted efficiency for six vapor deposition techniques.

| Method | Substrate | ETL-1 | ETL-2 | Perovskite | HTL-1 | HTL-2 | Back electrode | AX evaporation ratio (Å/s) | BX_2_ evaporation ratio (Å/s) | Annealing temperature (℃) | Annealing time (min) | pressure/mbar (10^-6) | thickness (nm) | Area (cm²) | PCE（%） |
| --- | --- | --- | --- | --- | --- | --- | --- | --- | --- | --- | --- | --- | --- | --- | --- |
| Single-source evaporation | FTO | SnO_2_ | None | Cs_0.04_FA_0.96_PbI_3_ | Spiro-OMeTAD | None | Au | 2.7 | 2.7 | 150 | 16 | 1.17 | 361 | 0.1 | 25.44 |
| Co-evaporation | FTO | SnO_2_ | None | Cs_0.04_FA_0.96_PbI_3_ | Spiro-OMeTAD | None | Au | 1.06 | 2.98 | 150 | 15 | 0.85 | 367 | 0.1 | 26.11 |
| Sequential vapor deposition | FTO | SnO_2_ | None | Cs_0.04_FA_0.96_PbI_3_ | Spiro-OMeTAD | None | Au | 1.00 | 2.97 | 148 | 14 | 1.5 | 366 | 0.1 | 26.21 |
| Evaporation-gas | FTO | SnO_2_ | None | Cs_0.04_FA_0.96_PbI_3_ | Spiro-OMeTAD | None | Au | / | 2.85 | 138 | 14 | / | 350 | 0.12 | 25.36 |
| Evaporation-solution | FTO | SnO_2_ | None | Cs_0.04_FA_0.96_PbI_3_ | Spiro-OMeTAD | None | Au | / | 2.75 | 150 | 15 | / | 370 | 0.06 | 25.76 |
| Solution-evaporation | FTO | SnO_2_ | None | Cs_0.04_FA_0.96_PbI_3_ | Spiro-OMeTAD | None | Au | / | / | 168 | 14 | / | 367 | 0.06 | 25.79 |

**Reference**

[1] A. El Bilali, A. Taleb, M. A. Bahlaoui, Y. Brouziyne, *J. Hydrol.* **2021**, 599, 126510.

[2] F. U. Kosasih, E. Erdenebileg, N. Mathews, S. G. Mhaisalkar, A. Bruno, *Joule* **2022**, 6, 2692.

[3] Y. Jiang, S. He, L. Qiu, Y. Zhao, Y. Qi, *Appl. Phys. Rev.* **2022**, 9, 021305.
